# *R-*gene variation across *Arabidopsis lyrata* subspecies: effects of population structure, selection and mating system

**DOI:** 10.1186/s12862-016-0665-5

**Published:** 2016-05-05

**Authors:** James Buckley, Elizabeth Kilbride, Volkan Cevik, Joana G. Vicente, Eric B. Holub, Barbara K. Mable

**Affiliations:** Institute of Biodiversity, Animal Health and Comparative Medicine, College of Medical, Veterinary and Life Sciences, University of Glasgow, Glasgow, G12 8QQ UK; School of Life Sciences, University of Warwick, Wellesbourne Campus, Wellesbourne, CV359EF UK; Current address: The Sainsbury Laboratory, Norwich Research Park, Norwich, NR47UH UK; Current address: Center for Adaptation to a Changing Environment, ETH Zurich, Zurich, 8092 Switzerland

**Keywords:** Disease resistance, Mating system, *Arabidopsis lyrata*, Selection, Inbreeding, Oomycete, Bacterial disease, Heterozygosity

## Abstract

**Background:**

Examining allelic variation of *R-*genes in closely related perennial species of *Arabidopsis thaliana* is critical to understanding how population structure and ecology interact with selection to shape the evolution of innate immunity in plants. We finely sampled natural populations of *Arabidopsis lyrata* from the Great Lakes region of North America (*A. l. lyrata*) and broadly sampled six European countries (*A. l. petraea*) to investigate allelic variation of two *R*-genes (*RPM1* and *WRR4*) and neutral genetic markers (Restriction Associated DNA sequences and microsatellites) in relation to mating system, phylogeographic structure and subspecies divergence.

**Results:**

Fine-scale sampling of populations revealed strong effects of mating system and population structure on patterns of polymorphism for both neutral loci and *R-*genes, with no strong evidence for selection. Broad geographic sampling revealed evidence of balancing selection maintaining polymorphism in *R*-genes, with elevated heterozygosity and diversity compared to neutral expectations and sharing of alleles among diverged subspecies. Codon-based tests detected both positive and purifying selection for both *R*-genes, as commonly found for animal immune genes.

**Conclusions:**

Our results highlight that combining fine and broad-scale sampling strategies can reveal the multiple factors influencing polymorphism and divergence at potentially adaptive genes such as *R*-genes.

**Electronic supplementary material:**

The online version of this article (doi:10.1186/s12862-016-0665-5) contains supplementary material, which is available to authorized users.

## Background

Natural variation in disease resistance conferred by pathogen specific *R-*genes has been essential for the evolution of innate immunity in plants [1–3]. The most common class of plant *R-*genes encodes receptor-like proteins containing a highly conserved nucleotide-binding site (NBS) and a variable leucine-rich repeat (LRR) region, which enable detection of effector proteins that are introduced into the host cytoplasm by an infecting pathogen. Balancing selection is predicted to be important in maintaining different resistance alleles to recognise diverse, coevolving pathogens [1, 3]. Characteristic signatures of balancing selection have been observed in many *R-*gene examples, including elevated levels of heterozygosity, nucleotide diversity and amino acid diversity, and reduced genetic structure across populations [4–6].

However, signatures of balancing selection were only detected in seven out of 27 loci in a comprehensive study of *R-*genes in *Arabidopsis thaliana* [4], suggesting that other types of selection or neutral demographic processes could affect allelic variation of plant *R*-genes. For example, genetic drift can be important in driving patterns of genetic polymorphism at adaptive loci in populations with fragmented distributions across landscapes [7]. Spatial and temporal heterogeneity in selection pressures from pathogens may maintain variation in *R-*genes, but make clear signatures of selection difficult to detect (e.g. [8]). Past demographic history and mating system variation could also substantially impact genetic variation at both neutral and adaptive loci. For example, population bottlenecks and postglacial recolonisation patterns have shaped genetic diversity patterns across species [9], and inbreeding is expected to reduce heterozygosity and diversity and increase differentiation among populations [10, 11]. In flowering plants, a shift from outcrossing to selfing is one of the most frequent evolutionary transitions [12], with selfing lineages, both within and between species, showing the predicted reduction in genetic variation [13–15]. However, balancing selection at *R-*genes could maintain elevated levels of heterozygosity and diversity in inbred lineages, as observed at animal immune genes [16, 17].

The current understanding of natural variation in plant *R-*genes has mostly been based on sampling one or a few plants (accessions) per population of the selfing, annual plant *A. thaliana* from across its range [1, 4, 18]. Recent studies of *R-*gene variation in *A. lyrata,* a close perennial relative of *A. thaliana,* revealed evidence for adaptive divergence at putative *R-*genes among geographically distant populations, but also an accumulation of major-effect mutations suggesting relaxed selection [19, 20]. The effects of mating system have also been considered in comparisons of other Brassicaceae species, where reduced variation was found in the selfing species *Capsella rubella* at five of nine studied *R-*genes when compared to its outcrossing relative *C. grandiflora* [21], with similar patterns of polymorphism at neutral loci [22]. However, interspecific comparisons of outcrossing and selfing taxa potentially confound mating system shifts with additional changes associated with speciation. Further investigation of how intraspecific variation in mating system may impact *R-*gene evolution could provide a more direct test of the relative impacts of mating system shifts and population structure on adaptive genetic diversity. For this, fine-scale sampling of local populations is important to understand the evolutionary processes shaping adaptive loci within and among populations [7]*.*

*Arabidopsis lyrata* is an herbaceous perennial that is predominantly outcrossing across its range. This species has distinct postglacial patterns of neutral polymorphism and structure across Europe (*A. l.* ssp. *petraea*) and North America (*A. l.* ssp. *lyrata*), with clear divergence between these lineages [20, 23, 24]. *A. l. petraea,* is found throughout Northern and Central Europe in small populations thought to represent glacial refugia [25], resulting in strong genetic structure within and among geographic regions, and particularly high levels of neutral genetic polymorphism in central European populations [20, 26, 27]. *A. l. lyrata* has strong population structure in Eastern North America [28–30] and reduced levels of polymorphism relative to *A. l. petraea* [20, 23]. Multiple transitions from outcrossing to selfing have been observed, particularly around the Great Lakes region, where populations vary from highly outcrossing to highly selfing [13, 29–32]. *A. lyrata* therefore provides the opportunity to assess how population structure at multiple scales (within and between subspecies) and mating system will impact variation at adaptive loci, such as *R*-genes.

In this study, we focused on two NBS-LRR proteins that confer resistance in *A. thaliana* to common biotrophic pathogens of the Brassicaceae: *RPM1* (Resistance to *Pseudomonas maculicola*) [33] and *WRR4* (White Rust Resistance) [34]. In *A. thaliana*, *RPM1* is a single copy gene for which functional alleles have been identified that recognise at least two different effector proteins of the bacterium *Pseudomonas syringae* [35]. This gene has been evolving by recurrent loss-of-function alleles, including major-effect mutations and deletion of the entire coding sequence [18, 33]. Variation in presence or absence of these alleles has been considered a balanced polymorphism and so the gene has been predicted to evolve under strong balancing selection [36]. Selection for multiple loss-of-function alleles suggests a cost of *RPM1-*mediated resistance in the absence of disease pressure [18, 37], despite *P. syringae* being a ubiquitous inhabitant of plant leaves [38, 39]. *WRR4* is one of three genes in a cluster that provides broad spectrum resistance in *A. thaliana* to white blister rust, caused by the biotrophic oomycete, *Albugo candida* [34]*. A. candida* is a global parasite that occurs as different pathotypes on brassica crops and wild Brassicaceae species (e.g. *Capsella bursa-pastoris* [40, 41]). However, there have been no previous studies of within or between population variation at *WRR4* so it has not been tested whether the gene is under balancing or diversifying selection.

To test whether selection acting at the resistance loci can be detected above demographic processes, we compared patterns of genetic variation at these two *R-*genes in *A. lyrata* to those observed at genome-wide Restriction Associated DNA (RAD) loci and neutral microsatellites. Moreover, we specifically asked whether intensive within-population sampling will alter perspectives on *R*-gene dynamics, compared to results from the broader-scale sampling used in most previous studies [4, 18]. We addressed two questions: 1) At a fine spatial scale, how does population structure, mating system variation and selection shape patterns of polymorphism within and among *A. l. lyrata* populations around the Great Lakes region? Specifically, we predicted that if the *R*-genes are under balancing selection this would result in higher heterozygosity and diversity, but reduced genetic structure among populations, relative to that observed at neutral microsatellites and RAD loci. Alternatively, directional selection at *R-*genes would reduce estimates of diversity and heterozygosity, and increase genetic differentiation, relative to neutral expectations. 2) At a broader geographic scale, how do subspecies divergence and selection shape patterns of polymorphism across subspecies of *A. lyrata*? Here, we predicted that balancing selection would result in increased allele sharing between the subspecies (trans-specific polymorphism) and that we would again observe an increase in heterozygosity and diversity but a reduction in genetic differentiation, relative to neutral expectations.

## Methods

### Sample collection and DNA extraction

**Fig. 1:**
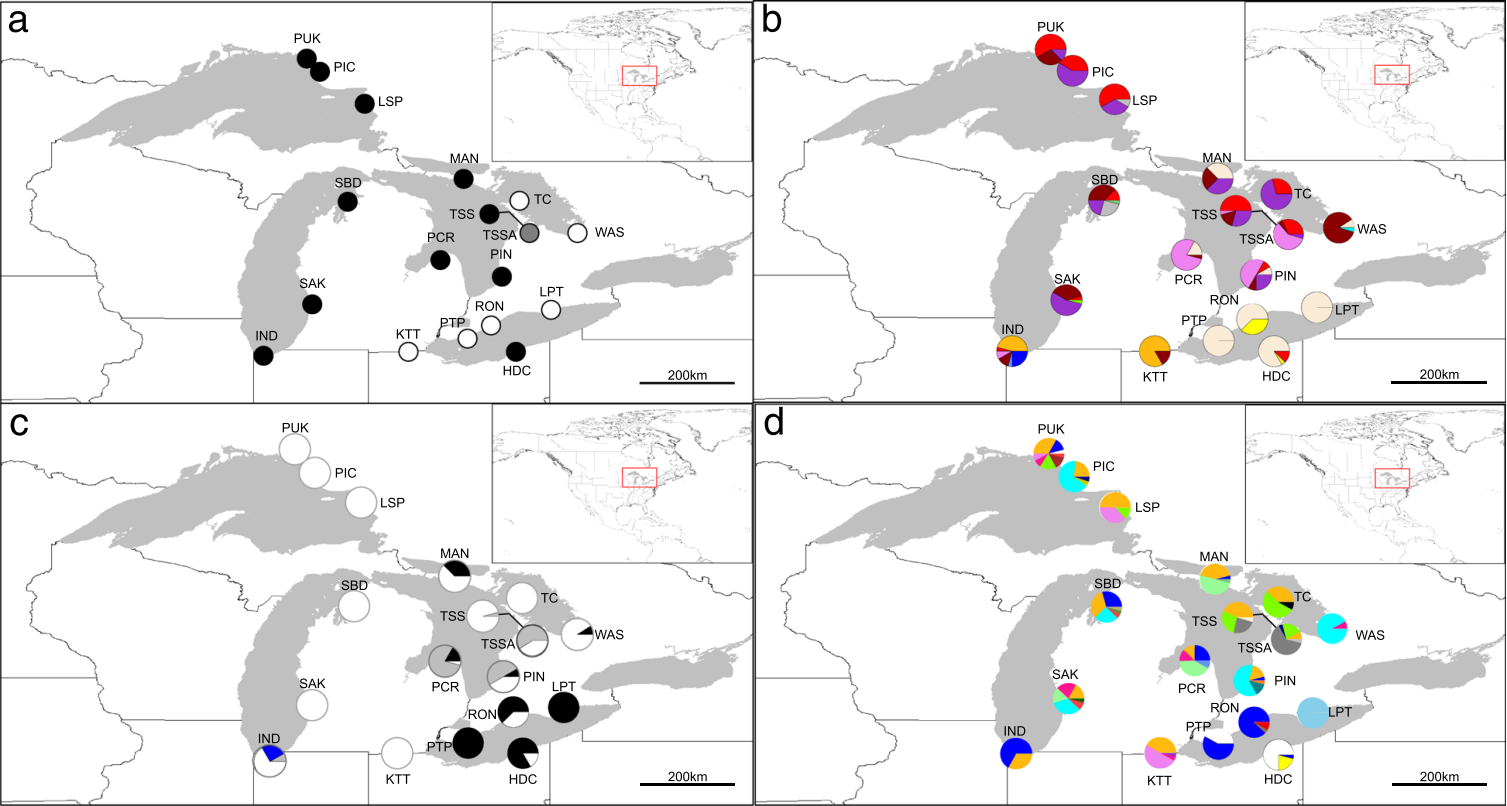
Maps of the distribution of *WRR4* and *RPM1* haplotypes in *A. l. lyrata* around the North American Great Lakes. Geographic distribution of 18 *Arabidopsis lyrata* ssp. *lyrata* sampling sites in the North American Great Lakes region showing variation in the dominant mating system at each site (based on previous estimates of outcrossing rates, T_m_ [28]) and the haplotype frequencies at two disease resistance loci (*WRR4* and *RPM1*): **a**) mating system variation, with black circles indicating outcrossing populations (T_m_ > 0.6), grey circles indicating a mixed-mating population (0.4–0.6) and white circles indicating selfing populations (T_m_ < 0.4); **b)** piecharts showing the frequency of the 13 haplotypes for *RPM1* (including a null allele); **c)** frequency of three *RPM1* haplotypes containing major effect mutations: a premature stop codon mutation (black), a null genotype (blue) and a 9 bp deletion (grey); all other haplotypes for which no major effect mutation was detected (white) and **d)**. piecharts showing the frequency in each population of the 29 haplotypes found for *WRR4* (see Table 1 for sample sizes), with each colour indicating a different haplotype

From the Great Lakes region, we focused on 18 diploid *A. l. lyrata* populations (Additional file 1: Table S1a) that show variation in mating system (Fig. 1a). In 2011, we sampled seven outcrossing (IND, MAN, PCR, PIN, SAK, SBD, TSS), two selfing (LPT, RON) and one mixed mating (TSSA) population (sample sizes in Additional file 1: Table S2a). To assess pathogen incidence, leaf tissue was sampled in early May when sand dune habitats are at their wettest and there is extensive green tissue available for pathogens to invade; the same sites were revisited in July to sample seeds. Samples were collected no closer than 5 m apart (to avoid sampling clones or family groups) along previously established transects [29]. Green leaf tissue collected in 2004 from a selfing population on the Bruce Peninsula (TC) was also included.

To extend the geographic range for *R*-gene sequencing, DNA extracted for a previous study [29] was included for seven other populations (outcrossing: HDC, LSP, PIC, PUK; selfing: KTT, PTP, WAS; Additional file 1: Table S2a). To allow comparison of neutral genetic variation with outcrossing rates based on a larger set of individuals per population, microsatellite genotypes (using loci described in [29]) were generated for the 2011 samples and data from the same set of loci [8] were used for the seven other populations (Additional file 1: Table S2a). For RAD genotyping, four individuals per population were grown from seed from each of the 10 populations sampled in 2011, as well as from three other selfing populations (PTP, KTT, TC) collected in other years (Additional file 1: Table S2a). Seeds were not available from HDC, PIC, PUK and WAS and therefore these populations were not genotyped using RAD-seq.

To assay variation across a broader-scale in *A. lyrata*, we obtained samples from diploid European populations collected by other researchers (Additional file 1: Table S1b). Samples were provided by Marcus Koch (dessicated leaf samples from Austria and Germany), Philippine Vergeer (seed samples from Scandinavian populations), and Elizabeth Bourne (seed and dessicated leaf samples from Scotland). Since few individuals were sampled per population and the aim was to compare variation with that in the Great Lakes region, sample sizes are reported per country (Additional file 1: Table S2b).

Seeds were germinated and grown in a growth cabinet (16:8 h day: night cycle; 20 °C: 16 °C; 80 % humidity), leaves sampled and then immediately dried using Drierite dessicant (WA Hammond Drierite Co. Ltd). Dried leaves were sent to the John Innes Centre (Norwich, UK), where DNA was extracted using the Qiagen DNeasy 96 plant kit (Qiagen Inc, Manchester, UK). For RAD sequencing, dried tissue was disrupted using a Fastprep machine and Fastprep lysing matrix A tubes (MP Biomedicals, Santa Ana, California, USA) and then DNA extracted using DNeasy spin columns. Multiple DNA extractions per individual were pooled to obtain the quantity of DNA required for library preparation. DNA quantification and quality checks were performed using the Nanodrop ND1000, Qubit 2.0 Fluorometer (Life technologies Ltd, Paisley, UK) and 2 % agarose gel electrophoresis.

### Pathogen frequency in *A. l. lyrata* populations around the North American great lakes

All 351 individuals from the 10 populations where leaves were field-collected in spring 2011 (sample sizes given in Additional file 1: Table S3) were screened for the presence of the pathogens associated with the two resistance genes, using a diagnostic PCR test (using primers and PCR conditions described in Additional file 1: Table S4). Any samples testing positive for a pathogen or showing faint bands (weak amplification) were re-tested to confirm presence. If the parasite-specific band was subsequently not amplified, then the test was repeated a third time to confirm presence/absence. In all cases, a negative PCR control was used, in addition to a positive control for that pathogen.

The presence of *Albugo sp.* (recognised by *WRR4*) was tested using primers published by Choi et al. [42], targeting the cytochrome *c* oxidase subunit II (COX2) region of mtDNA (see Additional file 1: Table S4 for primers and PCR conditions). A positive *A. candida* control isolate (AcEM2) from the University of Warwick, UK was added to each PCR run. The presence of a host-specific PCR product for the ITS primers was used as an internal control to ensure DNA was of good quality. The presence of *Pseudomonas* sp. (recognised by *RPM1*) was tested using Ps-for/Ps-rev primers [43], targeting a region of the 16 s rRNA gene specific to this bacterial genus (Additional file 1: Table S4). Any positive PCR bands were purified using ExoSAP-IT (Affymetrix, California, USA) and sent for direct sequencing (Edinburgh Genomics, Edinburgh, UK), with sequence identity confirmed by BLAST.

### *R*-gene sequencing

A fragment in the LRR region of *RPM1* was amplified using published primers [44] (primer sequences and PCR conditions are provided in Additional file 1: Table S4). For *WRR4,* primers were designed from conserved regions in exons 1, 2, 3 and 4 based on alignment of *A. thaliana* coding sequences with the corresponding region of two contigs extracted from the *A. l. lyrata* reference genome [45]. We compared variation at all four exons using a subset of the individuals used for RAD sequencing, along with sequences from a pilot study using additional North American sequences (a complete concatenated alignment, including the primer sequences, is available on DRYAD). Variation in exon 2 was consistent with the amplification of a single gene copy and heterozygotes could be resolved using direct sequencing due to lack of indels, so this exon was chosen for screening. By contrast, exon 1 showed evidence of amplification of paralogs, exon 3 was not polymorphic, and exon 4 (containing the LRR region) was highly polymorphic, but showed inconsistent amplification across samples.

PCR products were cleaned using ExoSAP-IT (Affymetrix, California, USA) and sent to Edinburgh Genomics (Edinburgh, UK) for sequencing in both directions (ABI3730 capillary sequencers). The strategy used to check sequence quality, resolve the phase for heterozygotes, and identify haplotypes is outlined in Supporting Information.

### Genome-wide SNP genotyping using RAD-seq

To set a neutral context for patterns of polymorphism and genetic structure for comparison with those resolved at the *R*-genes, we used single-end RAD sequencing data generated as part of a separate study for samples from Europe (*N* = 23) and the North America Great Lakes region (*N* = 49; sample details in Additional file 1: Table S2). Extracted DNA was sent to the sequencing facility, Edinburgh Genomics (Edinburgh, UK) for preparation of libraries for RAD-sequencing. Genomic DNA was digested using *Pst*I*,* a frequent cutting enzyme that is not sensitive to methylation. Each individual was tagged using barcodes differing by at least 2 bp. Digestion by *Pst*I was estimated to produce 64056 RAD loci for the 207 Mb *A. lyrata* genome. Three *Pst*I libraries were then generated for each of the two sample sets (49 *A. l. lyrata*; 23 *A. l. petraea*) and then each sequenced using one lane of an Illumina HiSeq 2000 sequencer at Edinburgh Genomics (Edinburgh, UK). Raw 100 bp reads were demultiplexed using their individual barcodes. FastQC (Babraham informatics) was used to check read quality within individuals and count total reads per individual. Reads were filtered to remove those with uncalled bases and those of low quality using *process_radtags* (*Stacks* v 1.30; [46]) and each read was trimmed to 92 bp to remove the ID tags.

Reads were aligned to the *Arabidopsis lyrata* ssp. *lyrata* reference genome (Phytozome version 107) using bowtie version 0.12.9 [47]. Default bowtie parameters were used to align reads to the eight chromosomes of *A. lyrata,* ~ 197Mbp [45], ignoring extrachromosomal regions (679 scaffolds, ~10 Mbp in size). Reads that aligned to more than one location were excluded from the alignment, so only those aligning uniquely to one location were retained. *Stacks* v1.30 [46] was then used to process the aligned reads, assemble RAD loci and identify SNPs across each of the 72 individuals. *Stacks* uses a multinomial-based likelihood model for identifying SNPs for diploid organisms [46].

To establish a neutral background set of loci, for which heterozygotes could confidently be called, only loci with a minimum coverage of 10 reads and present in all individuals across both Europe and North America were retained for analyses. Although this set may include loci under selection, we expected that these would represent a low proportion of the total, so observed patterns would represent the genome-wide background. This resulted in inclusion of 6721 loci that could be reliably compared across subspecies. The proportion of heterozygous loci (i.e. 92 bp RAD haplotypes) per individual was calculated by counting the number of RAD loci heterozygous for a 92 bp haplotype using the ‘*haplotypes.tsv’* output file produced by *Stacks*. Nucleotide diversity was estimated as the average number of nucleotide differences for all pairwise comparisons of sequences within a sample group (populations of *A. l. lyrata,* countries of *A. l.petraea*), averaged across all RAD loci and divided by the length of the RAD sequence (92 bp). This results in a statistic that is comparable to the estimate of nucleotide diversity produced by DnaSP for the *R-*genes [48].

### Presence-absence polymorphism genotyping in the *R*-genes

Since *R*-genes are often characterised by presence-absence polymorphisms, 11–42 samples per population from *A. l. lyrata* were screened for amplification of PCR products of the size predicted for each target gene. “Null” alleles were identified by the absence of a PCR product from an individual for a particular *R*-gene, but the presence of PCR products for the other *R*-gene and a host-specific ITS product (used for assaying Pathogen infection) in the same individual. The PCR was repeated twice for confirmation (or three times if two PCR results differed). Alternative primer sets for *RPM1* (RPM-F2 and RPM-R2; Additional file 1: Table S4) were designed and tested to confirm results.

### Analysing patterns of polymorphism and selection within *A. l. lyrata*

For each of the *R*-genes, individuals were classified as heterozygous or homozygous based on the whole sequence and the proportion of heterozygous individuals estimated for each population (average observed heterozygosity). Individuals showing no amplification products were classified as null homozygotes. Nucleotide diversity, defined as the average number of nucleotide differences per site between two sequences [48], across all sites and at synonymous and non-synonymous sites separately, was calculated within each population using DnaSP version 5 [49], with deletions and gaps excluded. Variation in the spatial distribution and frequency of *RPM1* and *WRR4* haplotypes were visualised as pie charts in relation to population location.

Tajima’s D statistic [50] was estimated for each population using DnaSP. A binomial test using R statistical software [51] was used to test for an excess of positive *D* values compared to negative across the 18 *A. l. lyrata* populations.

McDonald-Kreitman tests [52] were conducted in DnaSP to compare the ratio of nonsynonymous to synonymous substitutions between *A. l. lyrata* and *A. thaliana* (AT1G56510) (i.e. fixed differences between species) to the ratio of nonsynonymous to synonymous differences within *A. l. lyrata* (i.e. polymorphism within species). An excess of non-synonymous substitutions within species relative to between species would be consistent with balancing selection, whereas an excess of non-synonymous substitutions between species is a signature of directional selection.

Balancing selection is also predicted to produce weaker patterns of genetic structure at adaptive loci relative to that estimated for neutral loci, whereas directional selection is predicted to increase structure among populations [53, 54]. Mantel tests with 10,000 permutations were conducted using the R package “vegan” [55] to first test for associations between genetic and geographic distance among populations (isolation-by-distance) for microsatellites (all 18 N. American populations), RAD loci (only 12 N. American populations) and *R-*genes. Regressions of population-pairwise genetic differentiation for *R-*genes and neutral loci on pairwise geographic distance were then performed using the multiple regression on distance matrices function in the R package “ecodist” [56]. By estimating the slope of the regression line and its significance, we could test whether *R-*genes showed shallower slopes (i.e. weaker patterns of isolation-by-distance) than those estimated using eight microsatellites and RAD loci. Finally, Mantel tests were used to test whether pairwise F_ST_ at *R-*genes was associated with neutral pairwise genetic structure as estimated by microsatellites and RAD loci. For all pairs of populations we estimated pairwise F_ST_ [57] for the *R*-genes using DnaSP (excluding all gaps in pairwise comparisons); for the eight microsatellite loci using Genepop [58, 59], using the Weir & Cockerham [57] Fst estimator and for each RAD locus using *Stacks* v1.30 (based on Fst described in [57]) and averaging across all loci to produce an average pairwise F_ST_. GPS coordinates (Additional file 1: Table S1a) were converted to decimal degrees and population pairwise geographic distance (km) matrices were produced using the R package ‘fossil’ [60].

### Testing for effects of mating system in *A. l. lyrata*

We compared differences in individual heterozygosity estimated using eight microsatellites and RAD-seq to patterns observed at the two *R-*genes. Differences in individual heterozygosity between outcrossing and selfing populations (excluding mixed mating TSSA) were tested using generalised linear mixed effects models with a fixed factor of mating system and population as a random factor. As described in the methods, the same individuals were genotyped for both *R-*genes, but different individuals (from the same populations) were genotyped at microsatellite and RAD loci. Analyses were implemented using the R package ‘lme4’ [61] using a binomial error distribution for microsatellites and RAD loci (response variable = number loci heterozygous vs number homozygous) and the *R-*genes (individuals either heterozygous, ′1‵, or homozygous, ′0‵). A quasibinomial error distribution was used where over-dispersion was observed. Likelihood-ratio tests were used to test for a significant change in the log-likelihood of a model on removal of each factor of interest.

A Generalised Linear Model with Poisson error with fixed effect of mating system (outcrossing vs selfing) was used to test whether more haplotypes were observed in outcrossing than selfing populations.

In the absence of selection, relationships between outcrossing rate (*T*_*m*_) and observed heterozygosity (*H*_*o*_) or nucleotide diversity would be predicted to be similar for the *R*-genes and neutral markers. Linear regressions were therefore conducted to test whether estimates of *T*_*m*_ (from [29]) significantly predicted: 1) population-level average *H*_*o*_ for microsatellites, RAD loci and the two *R-*genes; 2) nucleotide diversity at RAD-seq loci and the *R-*genes; and 3) synonymous and non-synonymous nucleotide diversity for the *R-*genes.

### Analysing patterns of polymorphism and selection across subspecies

For assessment of broad-scale variation across subspecies, we compared *RPM1* and *WRR4* variation among *A. l. petraea* samples with a subset of the 11 outcrossing *A. l. lyrata* populations (using 6 individuals per population to make sample sizes more comparable with Europe). Haplotype networks were generated using an infinite site model in the Pegas R package [62]. Haplotype networks indicating relative frequencies were generated for each subspecies separately. To test for evidence of trans-specific polymorphism (as predicted by balancing selection [63]), a network not scaled by frequency was then used to visualise haplotype sharing among subspecies.

To confirm previous observations that *A. l. lyrata* shows lower diversity and heterozygosity than *A. l. petraea*, nucleotide diversity and heterozygosity for samples grouped by country of origin were calculated for the RAD loci (Additional file 1: Table S1b). Given that observed heterozygosity estimates averaged across RAD loci are not directly comparable to observed heterozygosity at a single *R*- gene sequence, we did not compare absolute values of observed heterozygosity, but rather focused on the relative differences in heterozygosity among the sample groups. Specifically, estimates for *R-*genes were compared to the RAD loci to examine whether *R-*gene diversity and heterozygosity is similarly reduced in *A. l. lyrata* relative to *A. l petraea*; assumed if they are evolving neutrally. The percentage difference in observed heterozygosity and nucleotide diversity for each European country relative to the *A. l. lyrata* outcrossing subset was also calculated for both *R-*genes and all RAD loci to visualise the relative differences between countries and loci. A GLM with binomial error (or quasibinomial if overdispersed) was also conducted to statistically test for reduced individual heterozygosity in *A. l. lyrata* relative to *A. l. petraea*. The change in model deviance on removal of the fixed effect of subspecies was tested using a likelihood ratio test.

To test for selection, the Datamonkey server (www.datamonkey.org), which implements statistical tests associated with the programme HyPhy [64], was used to conduct codon-based tests for selection using the *R-*gene haplotype alignments for both *A. l. petraea* and *A. l. lyrata*. Specifically, codons showing an excess of non-synonymous variants relative to synonymous variants (evidence for diversifying selection) or an excess of synonymous mutations (evidence for purifying selection) were used. The methods employed are tree-based methods that compare the rate of nonsynonymous changes per nonsynonymous site to synonymous changes per synonymous site such that ratios greater than one are indicative of positive selection and ratios less than one indicative of purifying selection. The program CodonTest [64] was first used to choose the best fitting substitution model and then three different codon-based tests were compared to identify signatures of selection (SLAC, FEL and REL; [65]). SLAC (single-likelihood ancestor) estimates the number of nonsynonymous and synonymous changes at each codon, FEL (fixed-effects likelihood) fits substitution rate models on a site-by-site basis and REL (random-effects likelihood) uses a distribution of substitution rates to model variation across sites. REL is the least conservative and so can provide an indication of sites potentially under selection. MEME (Mixed Effects Model of Evolution [66]) was also used to detect episodes of periodic selection across the phylogeny, in addition to diversifying selection. McDonald-Kreitman tests were also conducted for *A. l. petraea* compared to *A. thaliana*, for comparison with the *A. l. lyrata* results.

## Results

### Pathogen frequency in *A. l. lyrata* populations around the North American great lakes

PCR screening of host tissues from 351 individuals collected in the spring 2011 revealed that *Albugo sp.* were present at very low frequency in natural populations around the Great Lakes. Direct sequencing and BLAST revealed that just one individual in the SAK population, located towards the southern end of Lake Michigan (Additional file 1: Table S3), was infected with *Albugo*. The individual with *Albugo* showed no symptoms of disease infection (it had large green leaves).

*Pseudomonas*-specific PCR products were amplified at high frequency in the ten populations sampled in the spring of 2011 (present in 87.5–100 % of individuals sampled in these populations; Additional file 1: Table S3), but were detected at much lower frequency in the TC and TCA populations collected in July of 2004 (5.6 % and 15.4 % respectively; Additional file 1: Table S3). However, inferring potential selection pressures by pathogenic *Pseudomonas* sp. was complicated by the amplification of multiple products in the PCR reactions; cloning and sequencing indicated that the 16S region used was not variable enough to distinguish pathogenic, epiphytic and symbiotic *Pseudomonas* species. Of 51 samples collected in spring 2011 for which sequence quality was good, a wide range of *Pseudomonas* sp. were identified, with 23 samples containing sequences identified as *P. syringae,* but further identification of pathovars was not possible.

### Presence-absence polymorphism in the *R*-genes

*RPM1* sequence was amplified in a majority of samples (427 of 435 individuals), except for nine out of 40 individuals sampled from one of the outcrossing populations (IND; Additional file 1: Table S5). A second set of *RPM1* primers (Additional file 1: Table S4) was used to confirm null amplifications. *WRR4* sequence was amplified in all but three individuals from two neighbouring inbreeding populations (RON and LPT; Additional file 1: Table S5); these individuals also showed lack of amplification using primers located in exons 1 and 4. Individuals with no PCR amplification could reflect null alleles because *ITS2* and the other *R*-genes were successfully amplified from the DNA.

### Patterns of polymorphism and selection within *A. l. lyrata*

Amongst 215 *A. l. lyrata* samples, 12 *RPM1* haplotypes were identified which differed at 11 nucleotide sites, in addition to a 9 bp deletion at position 966–975 bp, across the 984 bp alignment. The number of *RPM1* haplotypes per population ranged from 1 to 6 (Fig. 1b; Table 1) and varied in frequency across populations (Additional file 1: Table S6a). Outcrossing populations had on average 2.1× more haplotypes than selfing populations (Table 1; GLM change in deviance = 4.945, df = 1, *p* = 0.026). Geographic clustering of haplotypes containing major effect mutations was observed, including: a haplotype containing the nine base-pair deletion at >50 % frequency in three of the four populations bordering Lake Huron (PCR, PIN and TSSA; Fig. 1c); and a haplotype with an in-frame stop-codon (TGG to TGA at position 701-703 bp) at >63 % frequency around Lake Erie (PTP, RON, LPT, HDC). Both haplotypes were observed at lower frequencies in other regions (Fig. 1c).

**Table 1:** Patterns of polymorphism within and among North American Great Lakes populations of *A. l. lyrata* at both *RPM1* and *WRR4*

Population	*T*_*m*_	Microsatellites	RAD		N	RPM1						WRR4					
		H_o_	π	H_o_		H_o_	Hap_total_	π	π_syn_	π_non-syn_	Taj D	Hap_total_	H_o_	π	π_syn_	π_non-syn_	Taj D
IND	0.99	0.396	0.0011	0.0748	12	0.250	6	0.0017	0.0039	0.0011	0.407	2	0.333	0.0010	0.0023	0.0007	1.609
PCR	0.98	0.208	0.0010	0.0747	12	0.417	3	0.0009	0.0008	0.0009	−1.102	5	0.833	0.0019	0.0052	0.0009	0.827
PUK	0.96	0.297	-	-	12	0.417	3	0.0014	0.0043	0.0006	1.773^a^	8	0.750	0.0033	0.0093	0.0016	0.797
LSP	0.94	0.156	-	-	12	0.167	3	0.0006	0.0022	0.0002	0.360	3	0.500	0.0013	0.0048	0.0003	0.346
SBD	0.94	0.427	0.0012	0.0883	12	0.917	5	0.0020	0.0050	0.0012	1.344	6	0.833	0.0025	0.0047	0.0018	−0.242
TSS	0.91	0.313	0.0010	0.0732	12	0.667	4	0.0011	0.0038	0.0004	0.922	5	0.667	0.0033	0.0090	0.0016	1.834^a^
SAK	0.9	0.375	0.0012	0.0776	12	0.583	4	0.0012	0.0027	0.0008	0.235	6	0.750	0.0017	0.0018	0.0017	−0.119
PIN	0.84	0.307	0.0009	0.0679	12	0.583	5	0.0011	0.0029	0.0006	−0.514	5	0.583	0.0015	0.0004	0.0018	−0.433
MAN	0.83	0.281	0.0009	0.0613	12	0.583	3	0.0023	0.0041	0.0018	1.972^a^	4	0.333	0.0009	0.0034	0.0002	−0.570
PIC	0.77	0.391	-	-	12^a^	0.500	2	0.0005	0.0024	0.0000	1.505	4	0.545	0.0009	0.0009	0.0009	−1.284
HDC	0.65	0.078	-	-	12	0.250	3	0.0008	0.0004	0.0009	−0.829	3	0.500	0.0007	0.0027	0.0001	−1.098
Outcrossing mean		0.294	0.0011	0.0740		0.485	3.73	0.0013	0.0029	0.0008		4.64	0.603	0.0017	0.0040	0.0011	
TSSA	0.41	0.198	0.0010	0.0671	12^b^	0.222	4	0.0004	0.0013	0.0001	−1.471	5	0.583	0.0027	0.0076	0.0013	1.634
KTT	0.31	0	0.0002	0.0062	12	0.000	2	0.0006	0.0013	0.0004	0.181	4	0.083	0.0009	0.0033	0.0002	−1.055
RON	0.28	0.042	0.0005	0.0291	12	0.417	2	0.0020	0.0023	0.0020	2.261^b^	3	0.250	0.0010	0.0030	0.0005	−0.875
WAS	0.25	0.094	-	-	12^b^	0.182	3	0.0010	0.0020	0.0007	−0.937	2	0.000	0.0004	0.0000	0.0005	−0.890
TC	0.18	0.042	0.0000	0.0015	12	0.083	2	0.0004	0.0020	0.0000	1.027	3	0.083	0.0026	0.0082	0.0010	1.422
LPT	0.13	0.045	0.0002	0.0064	11	0.000	1	0.0000	0.0000	0.0000	-	1	0.000	0.0000	0.0000	0.0000	-
PTP	0.09	0.078	0.0004	0.0038	12	0.000	1	0.0000	0.0000	0.0000	-	2	0.167	0.0022	0.0074	0.0007	2.446^b^
Selfing mean		0.050	0.0004	0.0190		0.114	1.83	0.0007	0.0013	0.0005		2.50	0.097	0.0012	0.0037	0.0005	
^a^ *N* = 11 for WRR4																	
^b^ *N* = 11 for RPM1																	

Patterns of polymorphism within and among North American Great Lakes populations of *A. l. lyrata* at both *RPM1* and *WRR4* and the averages for all outcrossing and all selfing populations. Populations ordered by population-level outcrossing rates (***T***_***m***_) from [28]. Observed heterozygosity (H_o_): average proportion heterozygous loci for microsatellites/RAD loci; proportion heterozygous individuals for *R-*genes. Total number of haplotypes (Hap_total_) observed at *RPM1* and *WRR4* within populations. Nucleotide diversity (π): average number pairwise differences divided by sequence length (as calculated by [47]). Synonymous/non-synonymous nucleotide diversity (π_syn/non-syn_) calculated using DnaSP v5 [48]. Significance of Tajima’s D values (Taj D) were calculated with DnaSP v5 and denoted by: ^a^ 0.05 < *p* < 0.1 and ^b^ *p* < 0.05. TSSA is classified as mixed mating, so was excluded from the average for selfing populations. ‘-‘indicates that data were not available (due to absence of samples for RAD-sequencing, or lack of within-population polymorphism for calculating Tajima’s D)

At *WRR4,* 29 haplotypes were observed amongst 215 individuals and these differed at 26 sites in the 909 bp alignment. This included one haplotype with a 2 bp deletion and another containing a mutation to a stop codon (from TTG to TAG at position 836 bp), which was homozygous in a single individual from the PCR population. The number of haplotypes per population ranged from 1 to 8 (Table 1) and limited regional clustering of alleles was observed (Fig. 1d), although one haplotype was shared at high frequency across multiple populations (Additional file 1: Table S6b). Outcrossing populations had on average 1.9× more haplotypes than selfing populations (Table 1; GLM change in deviance = 4.899, df = 1, *p* = 0.027).

Across the Great Lakes, the proportion of heterozygous individuals and nucleotide diversity varied extensively across populations but was generally similar between the two *R*-genes (Table 1). The selfing LPT population consistently showed the lowest nucleotide diversity and heterozygosity for both *RPM1* and *WRR4.* Synonymous nucleotide diversity was equal to or greater than non-synonymous diversity for 16 of the 18 populations for both *R-*genes. For *WRR4,* 9 of 18 populations had positive Tajima’s *D* values (Binomial test of deviation from 1:1 ratio: *p* = 1.00) and for *RPM1* 11 of 17 populations had positive *D* values (Binomial test: *p* = 0.332). Within populations, significantly positive D values were found only for PTP at *WRR4* and RON at *RPM1*, which suggests a significantly higher proportion of variants at intermediate frequency than expected based on neutral predictions. In most other populations the majority of haplotypes were observed at low frequency for both *RPM1* and *WRR4* (Additional file 1: Table S6). McDonald Kreitman tests suggested no evidence for an excess of non-synonymous substitutions within *A. l. lyrata* or between *A. l. lyrata* and *A. thaliana* at *RPM1* (G-value = 1.890, *p*-value = 0.169) or *WRR4* (G value = 0.015, *p* = 0.903).

No evidence for isolation by distance was observed with the microsatellites (*p* = 0.357; Additional file 1: Figure S2a), 6721 RAD loci (*p* = 0.108; Additional file 1: Figure S2b) or *WRR4* (*p* = 0.489; Additional file 1: Figure S2d), but *RPM1* showed a weakly significant association between genetic and geographic distance (*p* = 0.048; Additional file 1: Figure S2c). Regressions of *R-*gene genetic distance and geographic distance also showed that variation in geographic distance did not predict variation in genetic distance (slopes not significantly different from zero) for either of the *R-*genes (*RPM1*: *R*^2^ = 0.047, *p* = 0.0678; *WRR4*: *R*^2^ = 0, *p* = 0.991) or neutral loci (Eight microsatellites: *R*^2^ = 0.003, *p* = 0.7013; RAD loci: *R*^2^ = 0.044, *p* = 0.174). The slopes of the regression lines were similar for both *R-*genes (*RPM1:* y = 0.0003× + 0.342; *WRR4:* y = 0.0000× +0.384) and neutral loci (Eight microsatellites: y = 0.0001× + 0.463; RAD loci: y = 0.00004× + 0.060), suggesting selection is not shaping genetic structure among the North American Great Lakes populations.

**Fig. 2:**
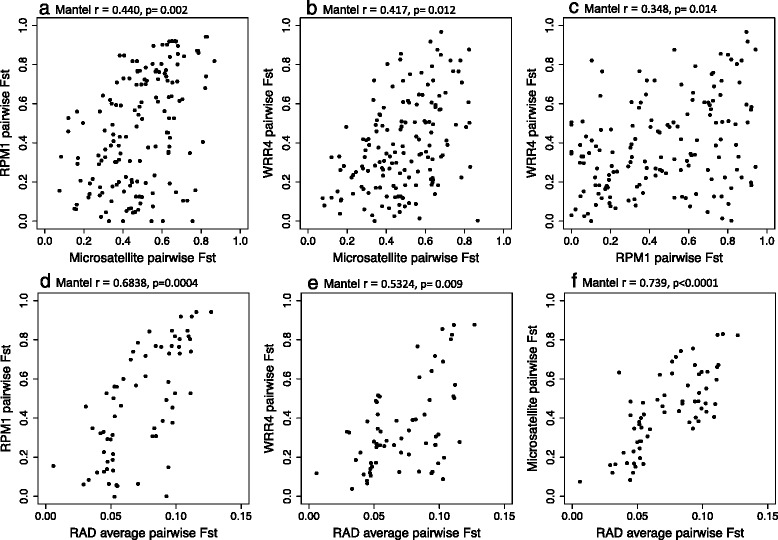
Pairwise genetic distance mantel correlations between microsatellites, 6721 RAD loci and both *R-*genes. Pairwise genetic distance mantel correlations between: **a**) *RPM1* and eight neutral microsatellites, **b**) *WRR4* and eight neutral microsatellites, **c)** *RPM1* and *WRR4,* **d)** *RPM1* and RAD loci, **e)** *WRR4* and RAD loci and **f**) RAD loci and eight neutral microsatellites in *A. l. lyrata*. The Mantel test statistic and p-value (based on 10,000) permutations are given

Pairwise F_ST_ for both *RPM1* and *WRR4* were significantly positively associated with pairwise genetic structure estimated using neutral microsatellites (*p* < 0.002; Fig. 2a, b); genetic structure at the two *R-*genes was also significantly correlated (*p* = 0.014; Fig. 2c). This result is supported using the 6721 RAD loci; pairwise genetic structure averaged across RAD loci (for a subset of 12 populations) was significantly correlated with genetic structure at both *R-*genes (*p* < 0.009; Fig. 2d, e), and with pairwise neutral genetic distance estimated by the eight microsatellites (*p* < 0.0001; Fig. 2f).

### Effects of mating system in *A. l. lyrata*

Average observed heterozygosity (*H*_*o*_*)* as estimated by microsatellites was 5.9× higher for outcrossing populations when compared to selfing populations (Table 1), as has been found previously [29]. Similarly, *H*_*o*_ was 7.9× higher in outcrossing populations across the 6721 RAD loci shared among all individuals, as well as 4.3× and 6.2× higher for *RPM1* and *WRR4* respectively, compared to selfing populations. By contrast, nucleotide diversity across RAD loci was 3.9× higher in outcrossing relative to selfing populations, but only 1.9× and 1.4× higher in outcrossing populations for *RPM1* and *WRR4,* respectively (Table 1).

GLM analyses showed significantly higher individual observed heterozygosity in outcrossing than selfing populations at both RAD and microsatellite loci (*p* < 0.0001; Additional file 1: Figure S1a, b), and at the two *R-*genes (*p* < 0.0005; Additional file 1: Figure S1c, d).

**Fig. 3:**
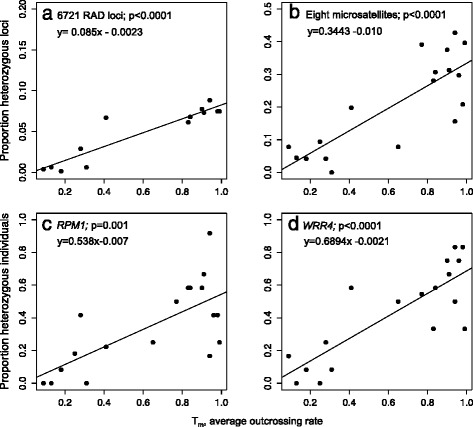
Associations between population-level outcrossing rates and observed heterozygosity at RAD loci and *R-*genes in *A. l. lyrata* Regressions of population-level outcrossing rates, T_m_ (from [28]) on observed heterozygosity in *A. l. lyrata* for: **a**) 6721 RAD loci; **b**) eight microsatellites; and two disease resistance genes, **c**) *RPM1* and **d**) *WRR4*. The significance (p-value) of the linear regression of population average outcrossing rate on observed heterozygosity is given, along with the linear equation describing the relationship

**Fig. 4:**
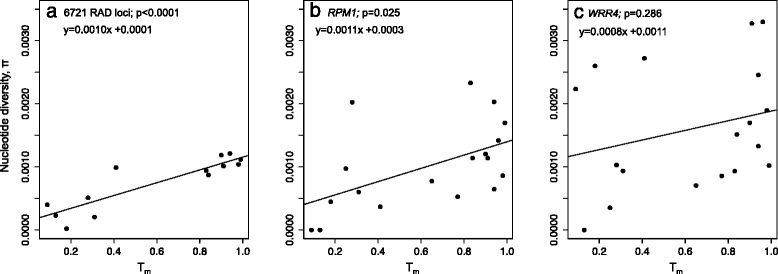
Associations between population-level outcrossing rates and nucleotide diversity at RAD loci and *R-*genes in *A. l. lyrata* Associations between population-level outcrossing rates, T_m_ (from [28]) and nucleotide diversity [47] in *A. l. lyrata*: **a**) averaged across 6721 RAD loci, **b**) for *RPM1* and **c**) for *WRR4*. The significance (*p*-value) of the linear regression of population average outcrossing rate on nucleotide diversity is given, along with the linear equation describing the relationship

Population outcrossing rates (*T*_*m*_) significantly predicted variation in observed heterozygosity for both RAD loci (F_1,11_ = 63.82, *p* < 0.0001, Adjusted R-squared = 0.840; Fig. 3a) and microsatellites (F_1,16_ = 31.19, *p* < 0.0001; Adjusted R-squared = 0.640; Fig. 3b). Similarly, outcrossing rates significantly explained a high proportion of the variation in population-level average *H*_*o*_ at *RPM1* (F_1,16_ = 15.85, *p* = 0.001; adjusted *R*^2^ = 0.466; Fig. 3c) and *WRR4* (*F*_1,16_ = 33.29, *p* < 0.0001; adjusted *R*^2^ = 0.655; Fig. 3d). Population-level outcrossing rates also significantly predicted variation in nucleotide diversity for RAD loci (*F*_1,11_ = 41.2, *p* < 0.0001; Adjusted *R*^2^ = 0.770; Fig. 4a) and *RPM1* (*F*_1,16_ = 6.101, *p* = 0.025; adjusted *R*^2^ = 0.231; Fig. 4b)*,* but not for *WRR4* (*F*_1,16_ = 1.218, *p* = 0.286; adjusted *R*^2^ = 0.0127; Fig. 4c). The same pattern was found when synonymous diversity was considered separately but outcrossing rates did not significantly predict variation in non-synonymous diversity at either *R-*gene (Additional file 1: Figure S3).

### Patterns of polymorphism and selection across subspecies

**Fig. 5:**
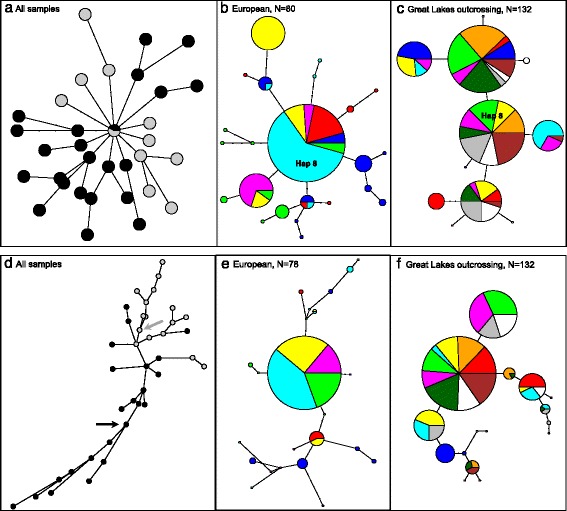
Haplotype networks in *Arabidopsis lyrata* produced by the Pegas R package [60] for *RPM1* (**a, b, c**) and *WRR4* (**d, e, f**) with circles either unscaled (**a, d**) or scaled by relative frequency (**b, c, e, f**). Black circles indicate European haplotypes and grey indicates North American (in **a** and **d**); coloured circles indicate different countries (*A. l. petraea*) or sampling sites (*A. l. lyrata*) in the other plots. N indicates the number of sequences included. The *RPM1* haplotype shared between European and Great Lakes samples (‘Haplotype 8’; Additional file 1: Table S6a) is indicated as ‘**Hap 8**’ in **b**) and **c**). In **d**) the black and grey arrows denote the most frequent haplotype as observed in Europe and Great Lakes respectively

Among the European samples (*A. l. petraea*), no null alleles were identified for *RPM1,* but four null genotypes were found for *WRR4* in the Austrian and German populations tested (Additional file 1: Table S5). For *RPM1*, three Norwegian samples contained an in-frame stop codon mutation (CAA to TAA) at position 661–663 bp but no major effect mutations were observed for *WRR4* in *A. l. petraea*. European samples added an additional 22 haplotypes for *RPM1* and 23 haplotypes for *WRR4,* but at *RPM1*, a high frequency allele found among the Great Lakes *A. l. lyrata* samples also occurred at high frequency (23 gene copies out of 80) in the *A. l. petraea* samples (‘Haplotype 8‵ on Fig. 5b, c). For *WRR4,* although there were no shared haplotypes between subspecies, there were single-step mutations derived in both directions (Fig. 5d).

*A. l. petraea* individuals had significantly higher observed heterozygosity (proportion heterozygous loci) at RAD-seq loci than *A. l. lyrata* outcrossing individuals (Table 2; *F* = 284.36, df = 1, *p* < 0.0001), with estimates of *H*_*o*_ >101.6 % higher in each European country relative to that observed in outcrossing *A. l. lyrata* (except Iceland, Additional file 1: Figure S4a). By contrast, *RPM1* showed no significant overall difference in *H*_*o*_ between the subspecies (Table 2; Change in deviance = 0.201, df = 1, *p* = 0.654). However, *H*_*o*_ at *RPM1* was >89 % higher in Austria and Germany relative to *A. l. lyrata*, (similar to the pattern at RAD loci), but consistently lower in Scandinavian countries and Scotland (Additional file 1: Figure S4a). Interestingly, for *WRR4* the proportion of heterozygous individuals was on average higher in outcrossing *A. l. lyrata* than in *A. l. petraea* samples (Table 2; Additional file 1: Figure S4; Change in deviance = 4.657, df = 1, *p* = 0.031). However, the German population showed particularly reduced levels of *WRR4* heterozygosity (Additional file 1: Figure S4a), possibly influenced by the presence of null alleles; when German samples were removed there was no difference between subspecies (*N* = 36, *H*_*o*_ = 0.389; Change in deviance = 3.275, df = 1, *p* = 0.0704).

**Table 2:** Observed heterozygosity and nucleotide diversity across European *A. l. petraea* and N. American *A. l. lyrata* outcrossing samples at 6721 RAD loci, *RPM1* and *WRR4*

	RAD loci			*RPM1*					*WRR4*				
Group	*N*	*H*_*o*_	π	*N*	*H*_*o*_	π	π_syn_	π_non-syn_	*N*	*H*_*o*_	π	π_syn_	π_nonpsyn_
Sweden	6	0.149	0.0025	6	0.500	0.0029	0.0038	0.0027	6	0.500	0.0007	0.0025	0.0002
Norway	7	0.154	0.0028	7	0.429	0.0021	0.0031	0.0018	7	0.429	0.0026	0.0069	0.0013
Iceland	3	0.132	0.0020	4	0.250	0.0003	0.0000	0.0003	4	0.500	0.0031	0.0089	0.0014
Scotland	2	0.155	0.0024	9	0.333	0.0009	0.0012	0.0008	11	0.273	0.0036	0.0100	0.0017
Austria	2	0.195	0.0033	9	0.889	0.0022	0.0051	0.0014	8	0.375	0.0055	0.0149	0.0028
Germany	3	0.223	0.0031	5	1.000	0.0021	0.0023	0.0020	3	0.000	0.0047	0.0104	0.0030
European average	23	0.162	0.0039	40	0.575	0.0028	0.0047	0.0022	39	0.359	0.0041	0.0108	0.0022
N. American Great Lakes outcrossing	28	0.074	0.0015	66	0.530	0.0019	0.0040	0.0013	66	0.576	0.0022	0.0056	0.0012

The samples size (N) for RAD-seq included 28 Great Lakes outcrossing individuals from seven populations and for both *R-*genes included 66 Great Lakes outcrossing individuals from 11 populations included (Additional file 1: Table S2 has details of sample locations). Observed heterozygosity (H_o_): Average proportion of heterozygous loci per individuals (RAD-seq) or proportion heterozygous individuals (*R-*genes). Nucleotide diversity (π, as calculated by [47]). For the RAD loci this is the average number of pairwise differences across 6721 loci divided by the length of the RAD sequence (92 bp). Synonymous/non-synonymous nucleotide diversity (π_syn/non-syn_) calculated using DnaSP version 5 [48]

For nucleotide diversity, variation at RAD loci was consistently higher *in A. l. petraea* than in *A. l. lyrata* (Table 2; Additional file 1: Figure S4b), with estimates of *π* >61.2 % higher in each European country relative to that observed in outcrossing *A. l. lyrata* (except Iceland, Additional file 1: Figure S4b). By contrast, nucleotide diversity at *RPM1* in European *A. l. petraea* populations was not consistently higher than in outcrossing *A. l. lyrata* (Additional file 1: Figure S4b; Table 2). Synonymous nucleotide diversity was higher than non-synonymous for the North American samples and all European countries, except Iceland (Table 2). At *WRR4,* nucleotide diversity was >15.9 % higher in *A. l. petraea* relative to outcrossing *A. l. lyrata*, except for samples from Sweden (Additional file 1: Figure S3b), which had 3.7× lower nucleotide diversity than observed for other European countries. Synonymous nucleotide diversity was at least 4.7× higher than non-synonymous diversity for all groups (Table 2). Nucleotide diversity values for both *R-*genes across all European samples and N. American outcrossing samples were similar to the mean nucleotide diversity across RAD loci for each group (Table 2) and fell within the frequency distribution of nucleotide diversity values for all RAD loci when plotted separately for all European samples and all N. American outcrossing samples (figure not shown).

For *RPM1,* positive (diversifying) selection was detected at one codon by both the REL and FEL methods and MEME detected an additional site under episodic diversifying selection (Additional file 1: Table S7a). However, there were 12 sites where negative selection was detected, with two found in all three analyses that tested this (REL, FEL, SLAC). For *WRR4* evidence for positive selection was found at two codons using both the REL and FEL methods (Additional file 1: Table S7b), but the MEME analyses suggested that one of these was actually under episodic selection (Additional file 1: Table S7b). Nine sites were found to be under purifying selection, with four detected by all three analyses.

McDonald Kreitman tests comparing *A. l. petraea* and *A. thaliana* showed that ratios of the number of synonymous and non-synsonymous mutations fixed or polymorphic within species did not significantly differ at *RPM1* (G-value = 1.673, *p* = 0.196). However, at *WRR4* there was evidence for significantly elevated proportion of non-synonymous mutations fixed between species (G-value = 5.178, *p* = 0.023).

## Discussion

Polymorphism at *R-*genes can be associated with resistance and susceptibility to particular pathogens, so it is important to understand how selection and neutral processes shape patterns of variation at these loci. To date most studies of *R-*gene variation in natural populations have focused on among population sampling of the annual, selfing plant *A. thaliana* [1]. However, mating system and local selection pressures can vary among taxa and populations and can shape patterns of neutral genetic variation [14, 22]. Finer-scale sampling of related taxa may therefore be important for identifying such effects on putatively adaptive genes. The results presented here highlight the complexity of factors influencing the evolutionary dynamics of plant *R*-genes in natural populations.

### Patterns of polymorphism and selection within *A. l. lyrata* (N. American subspecies)

For the North American populations sampled *(A. l. lyrata*), we found less presence-absence variation (PAV) at *RPM1* than expected based on genome-wide PAV patterns observed in *A. thaliana* [67, 68]. In *A. thaliana*, the fitness cost of a functional *RPM1* allele [37] has been predicted to maintain high frequencies of null alleles and major effect mutations at this locus [18, 36]. Interestingly, only one outcrossing population (IND) was found with individuals carrying a null *RPM1* allele (absence of an amplification product) at relatively high frequency (22 %). We also found two major effect *RPM1* mutations, which showed strong geographic structuring, with one occurring at more than 50 % frequency in three populations around Lake Huron and another occurring at more than 63 % frequency in four populations on Lake Erie (Fig. 1c).

The accumulation of mutations of putatively major effect on protein function has also been observed at two other *R-*genes in the IND and RON *A. l. lyrata* populations [19]. Whether this is due to a relaxation of pathogen-mediated selection pressure or balancing selection maintaining variation, is difficult to establish. In order to test this, we assessed prevalence of the target pathogens for each of the *R*-genes. Although we found *Pseudomonas* species to be present at high frequency across the Great Lakes (Additional file 1: Table S3), as observed in other natural plant populations (e.g. [39]), we could not determine the frequency of pathogenic isolates because of the lack of discrimination between species at the ITS locus used for detection. Additional PCR screening for the presence of pathogenic avirulence factors (e.g. [35]) may help identify potentially pathogenic *Pseudomonas*. However, the known pathogenic isolates of *Pseudomonas* in *A. thaliana* under laboratory conditions might be unable to cause symptoms and disease pressure in a natural environment on a related *Arabidopsis* species.

*WRR4* also shows PAV in *A. thaliana* (Cevik and Holub, unpublished data), but in *A. l. lyrata* we found null genotypes in only a few individuals from two selfing populations on Lake Erie. We expected the target pathogen, *Albugo candida,* to be present in the Great Lakes region, based on a previous report by Jacobson et al. [69] of widespread asymptomatic infection of *A. l. lyrata*. However, they found symptomatic infections only at SAK, where we also detected the pathogen in a single individual using the same PCR-based approach. High frequency of susceptibility in *A. l. lyrata* from North American sites to different pathovars of *A. candida* has been observed ([70]; Vicente and Holub, unpublished), which suggests that *WRR4* alleles able to recognise *Albugo* pathovars might be absent in this geographically defined subspecies of *A. lyrata*.

Sampling in one season prevents robust predictions about pathogen pressures and long-term dynamics of *R-*genes, but the results of our molecular genetic analyses are consistent with the absence of strong pathogen-mediated selection pressures for either of the target pathogens around the Great Lakes. Statistical tests for selection including Tajima’s D (based on haplotypes) or McDonald Kreitman tests (based on nucleotide polymorphisms) also showed no evidence for selection in *A. l. lyrata* and an excess of synonymous compared to nonsynomymous mutations in most populations (Table 1). Furthermore, for both *R*-genes we found that population pairwise F_ST_ were significantly associated with genetic structure at neutral markers (Fig. 2a, b, d, e), suggesting that neutral population genetic structure, specifically postglacial expansion patterns, has played a stronger role than balancing or directional selection in shaping patterns of variation at these *R-*genes in *A. l. lyrata*.

### Effects of mating system in *A. l. lyrata*

Balancing selection predicts the maintenance of higher levels of diversity and heterozygosity at adaptive loci in inbred lineages than observed at neutral markers [16, 17], as observed at two of nine *R-*genes in the selfing *Capsella rubella* [21]. For N. American *A. l. lyrata* we found that observed heterozygosity at RAD loci, neutral microsatellites and the *R-*genes were significantly explained by variation in population outcrossing rates (Fig. 3; Additional file 1: Figure S1). Outcrossing populations also tended to have a higher number of haplotypes than selfing populations (Table 1). Interestingly, nucleotide diversity was significantly explained by outcrossing rates at RAD loci and *RPM1* (Fig. 4a, b)*,* but not *WRR4* (Fig. 4c), with high polymorphism maintained in several of the inbred populations (Table 1). This could reflect the postglacial colonisation of this region by multiple lineages with divergent haplotypes independent of the dominant mating system, as suggested previously [28, 29], but may also be consistent with balancing selection. Since the Great Lakes region was colonised within the last 10,000 years, recent bottlenecks and gene flow among lineages arising from independent colonisation events could reduce signatures of selection. Furthermore, a lack of variation in inbred lineages might reduce the potential to detect differences in polymorphism resulting from selection. We therefore added a broad-scale perspective to identify signatures of selection by comparing *A. l. lyrata* with geographically distinct European populations.

### Patterns of polymorphism and selection across subspecies

*A. l. petraea* shows clear divergence from *A. l. lyrata* [20, 24] and distinct postglacial patterns of neutral polymorphism and structure have been observed among geographic regions [26, 27]. This was supported by our genome-wide RAD sequencing data, which showed higher diversity in *A. l. petraea* populations relative to outcrossing *A. l. lyrata* (Table 2; Additional file 1: Figure S3). Higher levels of polymorphism in *A. l. petraea* should increase our power to test for signatures of selection on the *R-*genes. Comparing subspecies also allows us to test for “trans-subspecific” polymorphism, in terms of allele sharing among divergent lineages [63, 71, 72]. Our results were broadly consistent with balancing selection: 1) patterns of polymorphism at the *R-*genes showed less of a distinction between the subspecies than observed for RAD loci (Table 2; Additional file 1: Figure S4), which results from higher levels of observed heterozygosity and nucleotide diversity in outcrossing *A. l. lyrata* than expected; and 2) the sharing of a high frequency haplotype between European and North American populations for *RPM1* (Fig. 5a). Although no haplotypes were identical between regions for *WRR4*, there were several haplotypes that only differed by a single base pair (Fig. 5d). Together, these results suggest that broad-scale sampling across subspecies identified clearer signatures of selection than finer-scale sampling in a limited geographic region. Our sampling in Europe was comparable to previous broad-scale studies in *A. thaliana* (e.g. [1, 4]), suggesting that sampling more populations with fewer individuals per population might be most informative for identifying selection.

Tests for selection based on nucleotide polymorphism and comparisons to *A. thaliana* (McDonald Kreitman test) did not show significant deviations from neutrality for *RPM1,* but did show a significant excess of non-synonymous polymorphisms fixed between species for *WRR4,* which is a signature of adaptive fixation of advantageous mutations [52]. For both *RPM1* and *WRR4,* codon-based tests for selection identified two sites under positive selection and a greater number of codons under purifying selection (Additional file 1: Table S7). Within *A. l. petraea,* the observed excess of synonymous diversity in all polymorphic populations (except Iceland for *RPM1;* Table 2) also suggests an important role of purifying selection at these loci*.* Pathogen presence was not screened for the European populations used here, because leaf samples were obtained at the same time as seed collections when presence of pathogens might be expected to be lower, because there is more investment in reproduction than in vegetative growth. By contrast, leaves from the North American populations were sampled during the wet part of the spring, to maximise chances of pathogen detection, and seeds were sampled during the dry part of the summer. Nevertheless, we have observed *Albugo* in the field in some of the European populations, whereas this has not been observed for the North American populations (Mable, personal observation). Moreover, high levels of resistance to different pathovars of *A. candida* has been observed in *A. l. petraea* relative to *A.l. lyrata* (Vicente and Holub, unpublished). The different conclusions based on sampling within North America and within Europe emphasizes the importance of considering differences in local selection pressures when testing patterns of variation in *R-*genes.

At immune genes in animals, such as the MHC, signatures of diversifying (positive) selection, such as high non-synonymous diversity, are often restricted to particular codons involved in antigen recognition, with high conservation of amino acids in the rest of the gene, resulting in the entire gene sequence showing no significant signatures of selection [73–75]. This emphasises the importance of using site-specific tests to assess evidence for positive selection, as most genes will include a combination of selected and conserved sites. Specific antigen binding sites at the MHC are conserved among vertebrates and can be used to test for selection specifically at these sites (e.g. [76]). However, there have been fewer comparative analyses of *R*-genes in plants to predict which sites will be involved in pathogen recognition across taxa. Previous studies of *R-*gene evolution in *A. thaliana* have focused on LRR regions to identify signatures of selection [4, 77], as these regions are involved in the recognition of corresponding pathogen avirulence products. It is thus intriguing that we found evidence for positive selection at *WRR4*, as well as *RPM1*, since we only obtained reliable sequences from the LRR region for the latter. The exon 2 region should span the NB-ARC domain of WRR4 [34], part of whose function is in nucleotide binding [78], but there have not been enough functional studies to determine whether there might be sites that bind to pathogen antigens in this region. Strong balancing selection is predicted to result in signatures of selection in sites linked to the actual regions involved in recognition processes, but this would be expected to affect multiple sites (Charlesworth [14]). Comparing variation between exons 2 and 4 (which spans the LRR region) for a subset of individuals used for RAD sequencing for European, North American outcrossing and North American selfing populations revealed quite similar patterns (Additional file 1: Table S8); an overall excess of synonymous diversity (particularly in North America) and no direct evidence of selection based on population-level tests. Although there was slightly weaker population differentiation based on exon 4 than exon 2 (Additional file 1: Table S8b), there was still substantial genetic structure among the three population sets and no allele sharing among European and North American samples. Codon-based tests revealed no sites under positive selection for exon 2 but nine sites under negative selection. The reduced evidence for positive selection compared to the full dataset (Additional file 1: Table S7b) could have been due to the absence of central European populations, which showed the highest divergence from the other populations. This was due to lack of amplification for exon 4. Exon 4 showed no evidence for positive selection but two codons showed episodic selection (Additional file 1: Table S9b) and 12 sites showed negative selection. Thus, while sequencing the LRR region would have been predicted to show stronger evidence of balancing selection, we did not find strong evidence for this based on these analyses.

## Conclusions

Overall, using both fine- and broad geographic sampling in *A. lyrata* revealed evidence for the effects of selection, mating system and population genetic structure on patterns of polymorphism and divergence at *R*-genes. Although we found little evidence for selection using fine-scale sampling within *A. l. lyrata*, this approach revealed low variation at *R-*genes in selfing relative to outcrossing populations and signatures of recent postglacial expansion into the Great Lakes region. By contrast, broad-scale comparisons between subspecies revealed some evidence for balancing selection in codon-based tests, haplotype sharing across widely divergent populations and subspecies, and patterns of *R-*gene variation distinct from neutral expectations. Combining results from sampling at different spatial scales may therefore improve our understanding of the neutral and selective forces shaping polymorphism at genes associated with adaptation.

## Consent for publication

Not applicable.

## Availability of data

*R-*gene sequence data has been submitted to Genbank with the following accession numbers. *RPM1* genotypes (IUPAC coded): KR137720 - KR137969; *RPM1* haplotype sequences: KR137970 - KR138003; *WRR4* genotypes (IUPAC coded): KR138056 - KR138308; *WRR4* haplotype sequences: KR138004 - KR138055.

## Additional file

Additional file 1: Methods: Heterozygote resolution for *R-*genes (*WRR4* and *RPM1*). **Table S1.** Details of the geographic locations from which samples were obtained for North American *A. l. lyrata* and the European subspecies, *A. l. petraea* [79]. **Table S2.** Number of samples used for RAD-genotyping, R-gene sequencing and microsatellites. **Table S3.** Prevalence of pathogens associated with *RPM1* and *WRR4* detected in *Arabidopsis lyrata* ssp. *lyrata* samples. **Table S4.** Primer details for amplicons targeted for R-genes and pathogen screening. **Table S5.** Number of individuals screened for presence or absence of a *RPM1* or *WRR4* amplification product. **Table S6.** Frequency of *RPM1* and *WRR4* haplotypes within each *A. l. lyrata* population. **Table S7.** Results of codon-based selection tests on *RPM1* and *WRR4* exon 2 haplotypes as implemented in Datamonkey. **Table S8.** Comparison of patterns of polymorphism for *WRR4* exon 2 and exon 4 regions [80, 81]. **Table S9.** Results of codon-based selection tests on *WRR4* exon 2 and exon 4 haplotypes. **Figure S1.** Variation in individual heterozygosity among *A.l.lyrata* populations ordered by outcrossing rate. **Figure S2.** Pairwise geographic distance and genetic distance mantel correlations for microsatellites, RAD loci and both *R-*genes. **Figure S3.** Regressions of synonymous/non-synonymous nucleotide diversity and population-level outcrossing rates in *A. l. lyrata*. **Figure S4.** Relative difference in observed heterozygosity between different *A. l. petraea* sample groups and *A. l. lyrata*. (DOCX 698 kb).
