# Supplementary material for: R-gene variation across Arabidopsis lyrata subspecies: effects of population structure, selection and mating system
Source: BMC Evol Biol. 2016 May 5;16:93. doi: 10.1186/s12862-016-0665-5 (PMC4858910; doi:10.1186/s12862-016-0665-5)
Supplement: Additional file 1: — Methods: Heterozygote resolution for R-genes (WRR4 and RPM1). Table S1. Details of the geographic locations from which samples were obtained for North American A. l. lyrata and the European subspecies, A. l. petraea [79]. Table S2. Number of samples used for RAD-genotyping, R-gene sequencing and microsatellites. Table S3. Prevalence of pathogens associated with RPM1 and WRR4 detected in Arabidopsis lyrata ssp. lyrata samples. Table S4. Primer details for amplicons targeted for R-genes and pathogen screening. Table S5. Number of individuals screened for presence or absence of a RPM1 or WRR4 amplification product. Table S6. Frequency of RPM1 and WRR4 haplotypes within each A. l. lyrata population. Table S7. Results of codon-based selection tests on RPM1 and WRR4 exon 2 haplotypes as implemented in Datamonkey. Table S8. Comparison of patterns of polymorphism for WRR4 exon 2 and exon 4 regions [80, 81]. Table S9. Results of codon-based selection tests on WRR4 exon 2 and exon 4 haplotypes. Figure S1. Variation in individual heterozygosity among A.l.lyrata populations ordered by outcrossing rate. Figure S2. Pairwise geographic distance and genetic distance mantel correlations for microsatellites, RAD loci and both R-genes. Figure S3. Regressions of synonymous/non-synonymous nucleotide diversity and population-level outcrossing rates in A. l. lyrata. Figure S4. Relative difference in observed heterozygosity between different A. l. petraea sample groups and A. l. lyrata. (DOCX 698 kb). [file 12862_2016_665_MOESM1_ESM.docx]

Article title: ***R-*gene variation across *Arabidopsis lyrata* subspecies: effects of population structure, selection and mating system**

Authors: James Buckley, Elizabeth Kilbride, Volkan Cevik, Joana G Vicente, Eric B Holub and Barbara K Mable

**SUPPORTING INFORMATION**

**METHODS**

**Heterozygote resolution for *R*-genes (*WRR4* and *RPM1*)**

Sequences were aligned, visually checked for false base assignment using Sequencher 4.7 (Gene Codes) and IUPAC ambiguity codes used to record heterozygous positions. Popset alignments of both the genotypes including IUPAC codes (RPM1: KR137720-KR137969, Popset: 937501658; WRR4 exon 2: KR138056-KR138308, Popset: 937502282) and haplotypes (RPM1: KR137970-KR138003, Popset: 937502115; WRR4 exon 2: KR138004-138308, Popset: 937502181) have been deposited to GenBank. Heterozygotes were resolved into haplotypes by eye. Briefly, sequences exported from Sequencher were aligned by hand using Se-Al v 2.0 ([Rambaut 1996](#_ENREF_3)) and then imported into MacClade version 4.06 ([Maddison and Maddison 2003](#_ENREF_2)). When possible, unique haplotypes were identified based on homozygotes and used to resolve heterozygotes. The following strategy was taken for resolving the phase for heterozygotes: 1) within each population, the consensus sequence was determined and polymorphic sites were identified and assigned to putative haplotypes; 2) haplotypes within populations were then compared across populations and homozygous sequences were used to confirm haplotypes resolved from the consensus approach; 3) for individuals with unresolved or ambiguous heterozygous genotypes, PCR products were cloned using TOPO TA Cloning Kits (Invitrogen Ltd, Paisley, UK) according to the methods described in Mable and Adam (2007); 4) haplotypes that were shared across populations were determined using the program Collapse, version 1.2 (http://darwin.uvigo.es/software/collapse.html); 5) gene copies within individuals were labeled a and b and complete genotype sequences for each individual were reconstructed using MacClade v. 4.06 (Maddison and Maddison 2003). To confirm heterozygote resolution we also used PHASE as implemented in DnaSP (Librado & Rozas, 2009).

**Supporting Information references:**

**Maddison DR, Maddison WP. 2003.** MacClade 4: Analysis of phylogeny and character evolution. Version 4.06. Sinauer Associates, Sunderland, Massachusetts.

**Rambaut A.** 1996. Se-Al: Sequence alignment editor, version 1.0 alpha 1.

**Table S1.** **Details of the geographic locations from which samples were obtained for North American *A. l. lyrata* and the European subspecies, *A. l. petraea****.* Summary of **a)** geographic location and average outcrossing rate (T_m_, from Foxe *et al.* 2010; SAK was described in Willi and Maattanen 2010) for each of the 18 populations sampled from the North American subspecies *A. l. lyrata,* and **b)** the sample collector and country of origin for 15 locations sampled and screened for *R-*genes from the European subspecies *A. l. petraea.* GPS coordinates and detailed site descriptions are available on request from the collector. All populations, except those marked by ‘*’, also had RAD genotyping data available for 2-3 individuals. Samples from the European population ‘Sjoviken’ were only RAD genotyped, neither *RPM1* or *WRR4* were sequenced for these individuals.

| Site | Location | State/Province, Country | Population GPS coordinates | | ***T_m_*** |
| --- | --- | --- | --- | --- | --- |
|  |  |  | Latitude | Longitude |  |
| ***a) Arabidopsis lyrata* subspecies *lyrata*** | |  |  |  |  |
| IND | Indiana Dunes National Lakeshore, Lake Michigan | Indiana, USA | N 41°37'17" | W 87°12'44" | 0.99 |
| PCR | Port Crescent State Park, Lake Huron | Michigan, USA | N 44°00'15" | W 83°04'26" | 0.98 |
| PUK | Oiseau Bay, Pukaskwa National Park, Lake Superior | Ontario, Canada | N 48°23'50" | W 86°11'34" | 0.96 |
| LSP | Gargantua Bay, Lake Superior Provincial Park, Lake Superior | Ontario, Canada | N 47°34'00 " | W 84°58'07" | 0.94 |
| SBD | Sleeping Bear Dunes National Lakeshore, Lake Michigan | Michigan, USA | N 44°56'20" | W 85°52'13" | 0.94 |
| TSS | Tobermory Singing Sands, BPNP, Lake Huron/Georgian Bay | Ontario, Canada | N 45°11'33" | W 81°35'02" | 0.91 |
| SAK | Saugatak Dunes State Park, Lake Michigan | Michigan, USA | N 42°42'164" | W 86°12'307" | 0.90 |
| PIN | Pinery Provincial Park, Lake Huron | Ontario, Canada | N 43°16'08" | W 81°49'53" | 0.84 |
| MAN | Manitoulin Island, Lake Huron/Georgian Bay | Ontario, Canada | N 45°40'13" | W 82°16'31" | 0.83 |
| PIC | Pic River First Nations Reserve, Lake Superior | Ontario, Canada | N 48°35'46" | W 86°18'09" | 0.77 |
| HDC | Headlands Dunes State Park, Lake Erie | Ohio, USA | N 41°45'43" | W 81°17'18" | 0.65 |
| TSSA | Tobermory Singing Sands Alvar, BPNP, Lake Huron/Georgian Bay | Ontario, Canada | N 45°11'27" | W 81°35'26" | 0.41 |
| KTT | Kitty Todd State Nature Preserve, Lake Erie | Ohio, USA | N 41°37'14" | W 83°47'15" | 0.31 |
| RON | Rondeau Provincial Park, Lake Erie | Ontario, Canada | N 42°15'41" | W 81°50'47" | 0.28 |
| WAS | Wasaga Beach Recreation Area, Georgian Bay | Ontario, Canada | N 44°30'59" | W 80°00'33" | 0.25 |
| TC | Tobermory cliffs, Bruce Peninsula National Park, Lake Huron/Georgian Bay | Ontario, Canada | N 45°14'30" | W 81°31'03" | 0.18 |
| LPT | Long Point Provincial Park, Lake Erie | Ontario, Canada | N 42°34'47 " | W 80°23'15" | 0.13 |
| PTP | Point Pelee National Park, Lake Erie | Ontario, Canada | N 41°55'40" | W 82°30'51" | 0.09 |

| Collector | Site | State/Province, Country |
| --- | --- | --- |
|  |  |  |
| ***b) Arabidopsis lyrata* supspecies *petraea*** | |  |
| Phillipine Vergeer | Hamnslatten | Sweden |
| Phillipine Vergeer | Norrfallsviken* | Sweden |
| Phillipine Vergeer | Notsand | Sweden |
| Phillipine Vergeer | Sjoviken | Sweden |
| Phillipine Vergeer | Bovra | Norway |
| Phillipine Vergeer | Laerdal | Norway |
| Phillipine Vergeer | Saebo | Norway |
| Phillipine Vergeer | Sandfell | Iceland |
| Phillipine Vergeer | Sandartunga | Iceland |
| Liz Bourne | Am Bodach* | Scotland |
| Liz Bourne | Beinn Dearg* | Scotland |
| Liz Bourne | Linn of Dee* | Scotland |
| Liz Bourne | Coyles of Muick | Cairngorms, Scotland |
| Marcus Koch | Veldensteiner Forst | Bavaria, Germany |
| Marcus Koch | Bad Vöslau | Lower Austria; South Vienna; Austria |
| Marcus Koch | Pernitz Pottenstein* | Lower Austria; Austria |

**Table S2: Number of samples used for RAD-genotyping, R-gene sequencing and microsatellites**. Numbers are summarised for the a) *A. l. lyrata* populations and b) *A. l. petraea* described in Table S1, indicating the date samples were collected and the sample type (leaves or seeds). *R-*gene PAV: number of samples screened for *R-*gene presence-absence variation; *RPM1/WRR4* sequencing: number of samples at which *RPM1* and *WRR4* were sequenced. PTP samples were collected in 2003 for microsatellites and *R-*genes, but in 2011 for RAD-seq genotyping, due to poor germination of older samples. Different individuals from the same family were used for RAD-seq genotyping than used for *R-*gene sequencing. Seeds were not available from HDC, PIC, PUK and WAS and therefore these populations were not genotyped using RAD-seq. Only one TC sample was successfully germinated for RAD-seq as seeds were collected in 2004. For *A. l. petraea*, sample sizes are given per country due to low sampling within populations.

| Site | Date collected | Tissue collected | Sample size | | | | |
| --- | --- | --- | --- | --- | --- | --- | --- |
|  |  |  | *R-*gene PAV | *RPM1* sequencing | *WRR4* sequencing | Microsatellites | RAD sequencing |
| ***a) A. l. lyrata*** | | |  |  |  |  |  |
| IND | 2011 | Leaves | 40 | 12 | 12 | 12 | 4 |
| PCR | 2011 | Leaves | 40 | 12 | 12 | 12 | 4 |
| PUK | 2003 | Seeds | 12 | 12 | 12 | 8 | - |
| LSP | 2004 | Seeds | 12 | 12 | 12 | 8 | - |
| SBD | 2011 | Leaves | 30 | 12 | 12 | 12 | 4 |
| TSS | 2011 | Leaves | 30 | 12 | 12 | 12 | 4 |
| SAK | 2011 | Leaves | 30 | 12 | 12 | 12 | 4 |
| PIN | 2011 | Leaves | 40 | 12 | 12 | 12 | 4 |
| MAN | 2011 | Leaves | 30 | 12 | 12 | 12 | 4 |
| PIC | 2003 | Seeds | 12 | 12 | 11 | 8 | - |
| HDC | 2007 | Seeds | 12 | 12 | 12 | 8 | - |
| TSSA | 2011 | Leaves | 24 | 11 | 12 | 12 | 4 |
| KTT | 2007 | Seeds | 12 | 12 | 12 | 8 | 4 |
| RON | 2011 | Leaves | 42 | 12 | 12 | 12 | 4 |
| WAS | 2003 | Seeds | 12 | 11 | 12 | 8 | - |
| TC | 2004 | Leaves | 31 | 12 | 12 | 12 | 1 |
| LPT | 2011 | Leaves | 14 | 11 | 11 | 11 | 4 |
| PTP | 2003 | Seeds | 12 | 12 | 12 | 8 | - |
|  | 2012 | Seeds | - | - | - | - | 4 |
| **Total** | |  | **435** | **213** | **214** | **187** | **49** |

| Country | Date collected | Tissue collected | Sample Size | | |
| --- | --- | --- | --- | --- | --- |
|  |  |  | *RPM1* sequencing | *WRR4* sequencing | RAD seq |
| ***b) A l.* *petraea*** | |  |  |  |  |
| Sweden | 2007 | Seeds | 6 | 6 | 6 |
| Norway | 2007 | Seeds | 7 | 7 | 7 |
| Iceland | 2007 | Seeds | 4 | 4 | 3 |
| Scotland | 2007/08 | Seeds | 9 | 11 | 2 |
| Germany | 2012 | Leaves | 5 | 5 | 3 |
| Austria | 2012 | Leaves | 10 | 10 | 2 |
| **Total** |  |  | **41** | **43** | **23** |

**Table S3**: **Prevalence of pathogens associated with *RPM1* and *WRR4* detected in *Arabidopsis lyrata* ssp. *lyrata* samples**. Prevalence of pathogens associated with *RPM1* (*Pseudomonas* sp.) and *WRR4* (*Albugo* sp) detected in *Arabidopsis lyrata* ssp. *lyrata* plants that were sampled around the North American Great Lakes. Populations ordered by decreasing outcrossing rates. The number of samples that gave a faint PCR band for *Pseudomonas* sp. are given in brackets.

| **Site** | **Collection year** | **Total** | **Positive for *A. candida*** | **Positive for *Pseudomonas* sp. (faint band)** |
| --- | --- | --- | --- | --- |
| IND | 2011 | 40 | 0 | 39 (3) |
| PCR | 2011 | 40 | 0 | 36 (7) |
| PUK | - | - | - | - |
| LSP | - | - | - | - |
| SBD | 2011 | 30 | 0 | 28 (10) |
| TSS | 2011 | 30 | 0 | 30 (9) |
| SAK | 2011 | 30 | **1** | 30 (10) |
| PIN | 2011 | 40 | 0 | 39 (4) |
| MAN | 2011 | 30 | 0 | 29 (5) |
| PIC | - | - | - | - |
| HDC | - | - | - | - |
| TSSA | 2011 | 24 | 0 | 21 (3) |
| KTT | - | - | - | - |
| RON | 2011 | 42 | 0 | 41 (6) |
| WAS | - | - | - | - |
| TC | 2007 | 31 | 0 | 3 (1) |
| LPT | 2011 | 14 | 0 | 14 (1) |
| PTP | - | - | - | - |

**Table S4**. **Primer details for amplicons targeted for R-genes and pathogen screening.** Primers, length of PCR products and PCR conditions are indicated. Since most of the target regions have extensive length polymorphisms, approximate amplicon sizes are indicated.

| **Name** | **Purpose** | **Direction** | **Sequence 5'-3'** | **Length** | **PCR reaction conditions** | **Reference** |
| --- | --- | --- | --- | --- | --- | --- |
| WRR4 exon2-F | Amplifies exon 2 of WRR4 gene | Forward | GAACGCCACACTGTCTAGGG | ~1000bp | 3 min at 94°C; 1 min at 56°C, 2 mins at 72°C, then 35 cycles of 30s at 94°C, 30s at 56°C, 2 mins at 72°C; then 6 mins at 72°C | Designed for paper |
| WRR4 exon2-R |  | Reverse | GAAGCAAATGGTGCATTACGAC |  |  |  |
| WRR4 exon4-F | Amplifies exon 4 (LRR) of WRR4 gene | Forward | CTCAAGGAGATGAATCTCGCT | ~1300bp | 3 min at 94°C; 1 min at 56°C, 2 mins at 72°C, then 35 cycles of 30s at 94°C, 30s at 56°C, 2 mins at 72°C; then 6 mins at 72°C | Designed for paper |
| WRR4 exon4-R |  | Reverse | GAGACAGCTATGGGAGCAGAG |  |  |  |
| RPM1F | Amplifies the LRR region of the gene RPM1 | Forward | GCAACAAACCTTCACTCTCTT | ~1100bp | 3 min at 94°C; 1 min at 52°C, 2 mins at 72°C, then 35 cycles of 30s at 94°C, 30s at 52°C, 2 mins at 72°C; then 6 mins at 72°C | Wang *et al.* 2011 |
| RPM1R |  | Reverse | ATGTGTTTAACCCTTGACCG |  |  |  |
| RPM1-F2 | Amplifies the LRR region of the gene RPM1 | Forward | CTTCTGAGAGCCTTAGACC | ~1000bp | 3 min at 94°C; 1 min at 52°C, 2 mins at 72°C, then 35 cycles of 30s at 94°C, 30s at 52°C, 2 mins at 72°C; then 6 mins at 72°C | Designed for paper |
| RPM1-R2 |  | Reverse | GGTCTACACTTCCATCTCC |  |  |  |
| albugocoII-F | Amplifies CoX2 region of mtDNA | Forward | GGCAAATGGGTTTTCAAGATCC | ~700bp | 3 min at 94°C; 50secs at 54°C, 1 min at 72°C, then 34 cycles of 30s at 94°C, 30s at 54°C and 1 min at 72°C; then 6 min at 72°C | Choi *et al.* 2006 |
| albugocoII-R |  | Reverse | CCATGATTAATACCACAAATTTCACTAG |  |  |  |
| ITS3 | Amplifies ITS2 region; General | Forward | GCATCGATGAAGAACGCAGC | 680bp | 4 min at 95°C; 35 cycles of 1 min at 95°C, 1 min at 56°C and 2 min at 72°C; then 4 min at 72°C | White *et al.* 1990; Protocol from Casimiro *et al.* 2004 |
| ITS4 |  | Reverse | TCCTCCGCTTATTGATATGC |  |  |  |
| Ps-for | Amplifies *Pseudomonas* specific 16S region | Forward | GGTCTGAGAGGATGATCAGT | 970bp | 5 min at 95°C; 30 cycles of 11s at 94°C, 15s at 92°C, 8s at 63°C, 1 min at 65°C, 10s at 74°C, 1min at 72°C; then 10 min at 72°C | Widmer *et al.* 1998 |
| Ps-rev |  | Reverse | TTAGCTCCACCTCGCGGC |  |  |  |

**Table S5**: **Number of individuals screened for presence or absence of a *RPM1* or *WRR4* amplification product**. Number of individuals screened within the North American Great lakes (*A. l. lyrata*) and Europe (*A. l. petraea*) are indicated. Total number of samples (N) screened for presence of a *RPM1* or *WRR4* allele are given, along with the number of null amplifications for each *R-*gene. One individual from TSSA showed poor amplification for ITS and *RPM1,* but we could get sequence for *WRR4*. One individual from Austria showed a length heterozygote genotype at *RPM1,* which was excluded from further analyses.

| **Location** | **Sample size** | | | |
| --- | --- | --- | --- | --- |
|  | ***RPM1 N*** | ***RPM1* null** | ***WRR4 N*** | ***WRR4* null** |
| ***A. l. lyrata*** | | |  |  |
| IND | 40 | 9 | 40 | 0 |
| PCR | 40 | 0 | 40 | 0 |
| PUK | 12 | 0 | 12 | 0 |
| LSP | 12 | 0 | 12 | 0 |
| SBD | 30 | 0 | 30 | 0 |
| TSS | 30 | 0 | 30 | 0 |
| SAK | 30 | 0 | 30 | 0 |
| PIN | 40 | 0 | 40 | 0 |
| MAN | 30 | 0 | 30 | 0 |
| PIC | 12 | 0 | 12 | 0 |
| HDC | 12 | 0 | 12 | 0 |
| TSSA | 24 | 0 | 25 | 0 |
| KTT | 12 | 0 | 12 | 0 |
| RON | 42 | 0 | 42 | 1 |
| WAS | 12 | 0 | 12 | 0 |
| TC | 31 | 0 | 31 | 0 |
| LPT | 14 | 0 | 14 | 2 |
| PTP | 12 | 0 | 12 | 0 |
| ***A. l. petraea*** | | |  |  |
| Sweden | 6 | 0 | 6 | 0 |
| Norway | 7 | 0 | 7 | 0 |
| Iceland | 4 | 0 | 4 | 0 |
| Scotland | 11 | 0 | 11 | 0 |
| Germany | 5 | 0 | 5 | 2 |
| Austria | 10 | 0 | 10 | 2 |

**Table S6: Frequency of *RPM1* and *WRR4* haplotypes within each *A. l. lyrata* population**. For **a)** *RPM1* the frequency of a null haplotype present in three IND individuals is included along with the 12 sequence haplotypes; for **b)** *WRR4* haplotypes shared at high frequency across multiple populations as described in main text are indicated by ***^a^***.

**a) *RPM1***

| **Pop^n^** | ***RPM1* Haplotypes** | | | | | | | | | | | | |
| --- | --- | --- | --- | --- | --- | --- | --- | --- | --- | --- | --- | --- | --- |
|  | **1** | **2** | **3** | **4** | **5** | **6** | **7** | **8** | **9** | **10** | **11** | **12** | **Null** |
| HDC | 0.833 | 0.125 | 0.042 | 0 | 0 | 0 | 0 | 0 | 0 | 0 | 0 | 0 | 0 |
| IND | 0 | 0.042 | 0 | 0.458 | 0.125 | 0.083 | 0.042 | 0 | 0 | 0 | 0 | 0 | 0.25 |
| KTT | 0 | 0 | 0 | 0.833 | 0.167 | 0 | 0 | 0 | 0 | 0 | 0 | 0 | 0 |
| LPT | 1.000 | 0 | 0 | 0 | 0 | 0 | 0 | 0 | 0 | 0 | 0 | 0 | 0 |
| LSP | 0 | 0.583 | 0 | 0 | 0 | 0 | 0 | 0.333 | 0.083 | 0 | 0 | 0 | 0 |
| MAN | 0.375 | 0 | 0 | 0 | 0.25 | 0 | 0 | 0.375 | 0 | 0 | 0 | 0 | 0 |
| PCR | 0.167 | 0 | 0 | 0 | 0.042 | 0.792 | 0 | 0 | 0 | 0 | 0 | 0 | 0 |
| PIC | 0 | 0.417 | 0 | 0 | 0 | 0 | 0 | 0.583 | 0 | 0 | 0 | 0 | 0 |
| PIN | 0.083 | 0.083 | 0 | 0 | 0.083 | 0.5 | 0 | 0.25 | 0 | 0 | 0 | 0 | 0 |
| PTP | 1 | 0 | 0 | 0 | 0 | 0 | 0 | 0 | 0 | 0 | 0 | 0 | 0 |
| PUK | 0 | 0.583 | 0 | 0 | 0.292 | 0 | 0 | 0.125 | 0 | 0 | 0 | 0 | 0 |
| RON | 0.625 | 0 | 0.375 | 0 | 0 | 0 | 0 | 0 | 0 | 0 | 0 | 0 | 0 |
| SAK | 0 | 0.042 | 0 | 0 | 0.375 | 0 | 0 | 0.542 | 0 | 0.042 | 0 | 0 | 0 |
| SBD | 0 | 0.125 | 0 | 0 | 0.375 | 0 | 0 | 0.208 | 0.25 | 0 | 0.042 | 0 | 0 |
| TC | 0 | 0.292 | 0 | 0 | 0 | 0 | 0 | 0.708 | 0 | 0 | 0 | 0 | 0 |
| TSSA | 0 | 0.318 | 0 | 0 | 0.045 | 0.591 | 0 | 0.045 | 0 | 0 | 0 | 0 | 0 |
| TSS | 0 | 0.5 | 0 | 0 | 0.167 | 0.042 | 0 | 0.292 | 0 | 0 | 0 | 0 | 0 |
| WAS | 0.091 | 0 | 0 | 0 | 0.864 | 0 | 0 | 0 | 0 | 0 | 0 | 0.045 | 0 |

**b) *WRR4***

| **Pop^n^** | ***WRR4* Haplotypes** | | | | | | | | | | | | | | | | | | | | | | | | | | | | |
| --- | --- | --- | --- | --- | --- | --- | --- | --- | --- | --- | --- | --- | --- | --- | --- | --- | --- | --- | --- | --- | --- | --- | --- | --- | --- | --- | --- | --- | --- |
|  | **1** | **2** | **3^a^** | **4^a^** | **5** | **6** | **7** | **8** | **9** | **10** | **11** | **12** | **13** | **14** | **15** | **16** | **17** | **18** | **19** | **20** | **21** | **22** | **23** | **24** | **25** | **26** | **27** | **28** | **29** |
| HDC | 0.75 | 0.21 | 0.04 | 0 | 0 | 0 | 0 | 0 | 0 | 0 | 0 | 0 | 0 | 0 | 0 | 0 | 0 | 0 | 0 | 0 | 0 | 0 | 0 | 0 | 0 | 0 | 0 | 0 | 0 |
| IND | 0 | 0 | 0.67 | 0.33 | 0 | 0 | 0 | 0 | 0 | 0 | 0 | 0 | 0 | 0 | 0 | 0 | 0 | 0 | 0 | 0 | 0 | 0 | 0 | 0 | 0 | 0 | 0 | 0 | 0 |
| KTT | 0 | 0 | 0 | 0.42 | 0.50 | 0.04 | 0.04 | 0 | 0 | 0 | 0 | 0 | 0 | 0 | 0 | 0 | 0 | 0 | 0 | 0 | 0 | 0 | 0 | 0 | 0 | 0 | 0 | 0 | 0 |
| LPT | 0 | 0 | 0 | 0 | 0 | 0 | 0 | 1.00 | 0 | 0 | 0 | 0 | 0 | 0 | 0 | 0 | 0 | 0 | 0 | 0 | 0 | 0 | 0 | 0 | 0 | 0 | 0 | 0 | 0 |
| LSP | 0 | 0 | 0 | 0.50 | 0.38 | 0 | 0 | 0 | 0.13 | 0 | 0 | 0 | 0 | 0 | 0 | 0 | 0 | 0 | 0 | 0 | 0 | 0 | 0 | 0 | 0 | 0 | 0 | 0 | 0 |
| MAN | 0 | 0 | 0.04 | 0.42 | 0 | 0.00 | 0 | 0 | 0 | 0.50 | 0.04 | 0 | 0 | 0 | 0 | 0 | 0 | 0 | 0 | 0 | 0 | 0 | 0 | 0 | 0 | 0 | 0 | 0 | 0 |
| PCR | 0 | 0 | 0.25 | 0.13 | 0 | 0.13 | 0 | 0 | 0 | 0.42 | 0 | 0.08 | 0 | 0 | 0 | 0 | 0 | 0 | 0 | 0 | 0 | 0 | 0 | 0 | 0 | 0 | 0 | 0 | 0 |
| PIC | 0 | 0 | 0 | 0.23 | 0 | 0 | 0 | 0 | 0 | 0 | 0 | 0 | 0.68 | 0.05 | 0.05 | 0 | 0 | 0 | 0 | 0 | 0 | 0 | 0 | 0 | 0 | 0 | 0 | 0 | 0 |
| PIN | 0 | 0 | 0.04 | 0.17 | 0 | 0 | 0 | 0 | 0 | 0 | 0 | 0 | 0.63 | 0 | 0 | 0.13 | 0.04 | 0 | 0 | 0 | 0 | 0 | 0 | 0 | 0 | 0 | 0 | 0 | 0 |
| PTP | 0.42 | 0 | 0.58 | 0 | 0 | 0 | 0 | 0 | 0 | 0 | 0 | 0 | 0 | 0 | 0 | 0 | 0 | 0 | 0 | 0 | 0 | 0 | 0 | 0 | 0 | 0 | 0 | 0 | 0 |
| PUK | 0.04 | 0 | 0.13 | 0.33 | 0.08 | 0.08 | 0 | 0 | 0.17 | 0 | 0 | 0 | 0 | 0 | 0 | 0 | 0 | 0.13 | 0.04 | 0 | 0 | 0 | 0 | 0 | 0 | 0 | 0 | 0 | 0 |
| RON | 0 | 0 | 0.88 | 0 | 0 | 0 | 0 | 0 | 0 | 0 | 0 | 0 | 0 | 0 | 0 | 0 | 0 | 0 | 0 | 0.04 | 0.08 | 0 | 0 | 0 | 0 | 0 | 0 | 0 | 0 |
| SAK | 0 | 0 | 0 | 0.17 | 0 | 0.21 | 0 | 0 | 0 | 0.17 | 0 | 0 | 0.33 | 0 | 0 | 0 | 0 | 0 | 0.08 | 0 | 0 | 0.04 | 0 | 0 | 0 | 0 | 0 | 0 | 0 |
| SBD | 0 | 0 | 0.29 | 0.33 | 0 | 0 | 0 | 0 | 0 | 0 | 0 | 0 | 0.25 | 0 | 0 | 0 | 0 | 0 | 0.04 | 0 | 0 | 0 | 0.04 | 0.04 | 0 | 0 | 0 | 0 | 0 |
| TC | 0 | 0 | 0 | 0.38 | 0 | 0 | 0 | 0 | 0.54 | 0 | 0 | 0 | 0 | 0 | 0 | 0 | 0 | 0 | 0 | 0 | 0 | 0 | 0 | 0 | 0.08 | 0 | 0 | 0 | 0 |
| TSS | 0 | 0 | 0 | 0.38 | 0 | 0 | 0 | 0 | 0.25 | 0 | 0 | 0 | 0 | 0 | 0 | 0 | 0 | 0 | 0 | 0 | 0 | 0 | 0 | 0 | 0 | 0.21 | 0 | 0.04 | 0.13 |
| TSSA | 0 | 0 | 0 | 0.08 | 0 | 0 | 0 | 0 | 0.21 | 0 | 0 | 0 | 0 | 0 | 0.04 | 0 | 0 | 0 | 0 | 0 | 0 | 0 | 0 | 0 | 0 | 0.63 | 0.04 | 0 | 0 |
| WAS | 0 | 0 | 0 | 0 | 0 | 0.08 | 0 | 0 | 0 | 0 | 0 | 0 | 0.92 | 0 | 0 | 0 | 0 | 0 | 0 | 0 | 0 | 0 | 0 | 0 | 0 | 0 | 0 | 0 | 0 |

**Table S7**: **Results of codon-based selection tests on *RPM1* and *WRR4* exon 2 haplotypes as implemented in Datamonkey.** A significance threshold of 0.1 was set for all tests. Indicated are codons showing episodic diversifying selection (only tested by MEME), positive selection (tested by all), and negative selection (not tested by MEME) and their significance in each of the tests (S=significant evidence of selection, NS =non-significant)*.* Note that the overall conclusion is that both genes are predominantly under purifying (negative) selection, although each has some codons showing evidence for positive selection.

| a) *RPM1* |  |  |  |  |  |
| --- | --- | --- | --- | --- | --- |
| Codon | SLAC | FEL | REL | MEME | Inference |
| 45 | na | na | na | **S** | Episodic selection |
| 160 | NS | **S** | **S** | NS | Positively selected |
| 90, 314 | **S** | **S** | **S** | na | Negatively selected |
| 64, 95, 146, 224, 265 | NS | **S** | **S** | na | Negatively selected |
| 27, 58, 177, 206, 291 | NS | NS | **S** | na | Negatively selected |

b) *WRR4*-exon 2

| Codon | SLAC | FEL | REL | MEME | Inference |
| --- | --- | --- | --- | --- | --- |
| 242 | na | na | na | **S** | Episodic selection |
| 71, 242 | NS | **S** | **S** | NS | Positively selected |
| 10, 32, 38, 285 | **S** | **S** | **S** | na | Negatively selected |
| 174 | **S** | **S** | NS | na | Negatively selected |
| 54, 63, 163, 234 | NS | **S** | NS | na | Negatively selected |

**Table S8:** **Comparison of patterns of polymorphism for *WRR4* exon 2 and exon 4 regions.** Sequences were compared at the two exons for a subset of European, N. American outcrossing and N. American selfing samples that had been used for the RAD sequencing analyses to test whether not focusing on the LRR region (exon 4) would result in lower evidence for balancing selection. Individual sample sizes were as follows: European subset, N=10 (from 8 populations across Sweden, Norway, Iceland and Scotland); N. American outcrossing, N=11 (from 6 populations); N. American selfing, N=6 (from 3 populations). a) Patterns of polymorphism and signatures of selection conducted with DnaSP version 5. None of the estimates of Tajima’s D were significant. McDonald Kreitman tests, using *A. thaliana* as a reference, also were not significant (p>0.2) but exon 2 sequences for the European sample set were borderline significant (G value = 3.6571; p = 0.05602), comparable to the MK test results for the larger set of European sample *WRR4* sequences described in the main text. b) Population differentiation based on WRR4 exon 2 and exon 4 sequences. Indicated are the G_ST_ (Nei 1973) and F_ST_ (Hudson et al. 1992) estimates implemented in DnaSP for each comparison, with the overall estimates for each and the estimated number of migrants (Nm) based on Nei (1973).

a)

|  | **European samples** | | **N.American outcrossing** | | **N.American selfing** | |
| --- | --- | --- | --- | --- | --- | --- |
|  | **Exon 2** | **Exon 4** | **Exon 2** | **Exon 4** | **Exon 2** | **Exon 4** |
| **Number sequences** | 20 | 20 | 22 | 22 | 12 | 12 |
| **Length (bp)** | 912 | 1170 | 912 | 1167 | 912 | 1167 |
| **Number haplotypes** | 6 | 8 | 6 | 12 | 3 | 4 |
| **Nucleotide diversity (π)** | 0.00294 | 0.00202 | 0.0018 | 0.00477 | 0.00219 | 0.00571 |
| **Synonymous π** | 0.00866 | 0.0028 | 0.0033 | 0.00914 | 0.00559 | 0.01128 |
| **Nonsynonymous pi** | 0.00128 | 0.00179 | 0.00137 | 0.00354 | 0.0012 | 0.00415 |
| **Tajima's D** | -0.47625 | -1.08732 | -0.00171 | -0.2907 | 0.76657 | 1.86885 |

b)

|  |  | **WRR4-exon2** | | | **WRR4-exon4** | | |
| --- | --- | --- | --- | --- | --- | --- | --- |
| **Comparison** | | **G**_ST_ | **F**_ST_ | **Nm-hap** | **G**_ST_ | **F**_ST_ | **Nm-hap** |
| Europe | NA outcrossing | 0.15204 | 0.624 |  | 0.07158 | 0.502 |  |
| Europe | NA inbreeding | 0.18474 | 0.617 |  | 0.10831 | 0.438 |  |
| NA outcrossing | NA inbreediing | 0.04541 | 0.06 |  | 0.05782 | 0.248 |  |
| Overall |  | 0.16985 | 0.54201 | 1.22 | 0.10093 | 0.3956 | 2.23 |

**Table S9:** **Results of codon-based selection tests on *WRR4* exon 2 and exon 4 haplotypes.** The set of individuals used are the same as described in the legend for Table S8*.*  Note that the reduced sample size for *WRR4*-exon 2 altered conclusions compared to the full set, with less evidence for positive selection, no evidence for episodic selection, and not all the same sites showing negative selection (particularly in the REL analyses).

| a) *WRR4*-exon 2 RAD set |  |  |  |  |
| --- | --- | --- | --- | --- |
| Codon | SLAC | FEL | REL | Inference |
| 10, 32, 38, 174, 211, 285 | NS | **S** | **S** | Negatively selected |
| 163, 205, 296 | NS | NS | **S** | Negatively selected |

b) *WRR4*-exon 4 RAD set

| Codon | SLAC | FEL | REL | MEME | Inference |
| --- | --- | --- | --- | --- | --- |
| 238, 327 | na | na | na | **S** | Episodic selection |
| 226 | **S** | **S** | **S** | na | Negatively selected |
| 63, 70, 97, 376 | **S** | **S** | **S** | na | Negatively selected |
| 105, 158, 230, 275, 309, 328, 336 | NS | NS | **S** | na | Negatively selected |

**Figure S1**. **Variation in individual heterozygosity among *A.l .lyrata* populations ordered by outcrossing rate.**

Summary of heterozygosity within *Arabidopsis lyrata* populations, including: a) the proportion heterozygous loci at 6721 RAD loci; **b)** the mean observed heterozygosity (*H_o_*, +/- one standard error) within populations at eight microsatellites; c) the proportion of heterozygous individuals within populations at *RPM1;* and d) the proportion of heterozygous individuals per population at *WRR4*. Black filled bars indicate outcrossing populations and white bars indicate selfing populations. TSSA includes a mixture of self-incompatible and self-compatible individuals and so is considered mixed mating (grey). The sample size (N =) is given below each graph with ‘nd’ indicating samples from this population not genotyped by RAD-seq. The effect of mating system as tested with a mixed effect GLM with binomial error (fixed effect of mating system, random effect of population) is given in each graph. Given are the likelihood ratio test statistics (LR), the degrees of freedom (df), and the probability value (p) indicating the significance of a change in model log-likelihood on removing the factor of interest from the model.

**Figure S2: Pairwise geographic distance and genetic distance mantel correlations for microsatellites, RAD loci and both *R-*genes.**

Pairwise genetic distance and geographic distance (km) scatter plots for: **a**) eight neutral microsatellites, **b**) 6721 RAD loci, **c)** *RPM1*, **d)** *WRR4.* The Mantel test statistic and p-value based on 10000 permutations are given.

**Figure S3: Regressions of synonymous/non-synonymous nucleotide diversity and population-level outcrossing rates in *A. l. lyrata***

Regressions of synonymous and non-synonymous nucleotide diversity on population-level outcrossing rates, T_m_ (from Foxe *et al.* 2010) for synonymous nucleotide diversity at (a) *RPM1* and (b) *WRR4,* and non-synonymous nucleotide diversity at (c) *RPM1* and d) *WRR4*. The F-statistic and significance (p-value) of the linear regression of outcrossing rate on the nucleotide diversity estimate is given, along with adjusted R-squared.

**Figure S4**: **Relative difference in observed heterozygosity between different *A. l. petraea* sample groups and *A. l. lyrata.***

Barplots showing the % difference in **(a)** observed heterozygosity and **(b)** nucleotide diversity for each European country relative to N. American *A. l. lyrata* outcrossing individuals (N=66). Black bars represent the estimate from 6721 RAD loci, grey bars represent *RPM1* and white bars represent *WRR4.*
